# Reactivity of mammalian lipoxygenases (ALOX isoforms) with phospholipids, biomembranes and lipoproteins

**DOI:** 10.1038/s42003-026-10233-9

**Published:** 2026-06-08

**Authors:** Xin Chen, Sarah Melissa Strätker, Sahanawaz Parvez, Ramunas Martin Vabulas, Astrid Bochert, Michael Rothe, Hermann-Georg Holzhütter, Polamarasetty Aparoy, Hartmut Kuhn

**Affiliations:** 1 Charité - Universitätsmedizin Berlin, Corporate member of Freie Universität Berlin and Humboldt Universität zu Berlin, Department of Biochemistry, Berlin, Germany; 2 Molecular Modeling and Protein Engineering Lab, Biology Division, Department of Humanities and Sciences, Indian Institute of Petroleum and Energy, Visakhapatnam, India; 3 Lipidomix GmbH, Berlin, Germany

**Keywords:** Phospholipids, Molecular medicine

## Abstract

Arachidonic acid lipoxygenases (ALOX-isoforms) have been implicated in cell differentiation and in the pathogenesis of various diseases. Human ALOX-isoforms prefer free polyunsaturated fatty acids as substrate but some of them are also capable of oxygenating complex ester lipids. Here we compared the reactivity of mammalian ALOX isoforms with complex lipid structures, explored the chemistry of the oxygenation products and characterized the structure of the enzyme-substrate complexes. We found that human and mouse ALOX15 orthologs as well as human ALOX15B are capable of oxidizing complex substates in the absence of adapter proteins and that the patterns of oxygenation products were similar to those of free fatty acid oxygenation. In contrast, the corresponding activities of mouse Alox15b and human ALOX12 were limited. Specific lipoxygenase products were also detected in the plasma lipids of mice with modified *ALOX15* gene suggesting the in vivo activity of the enzyme on complex ester lipid substrates.

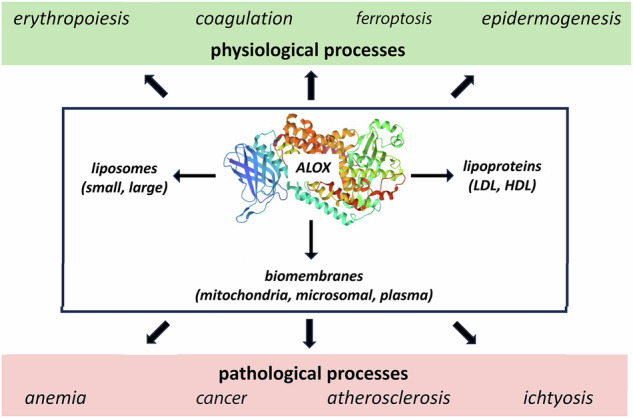

## Abstract (web summary)

Mammalian lipoxygenase isoforms oxygenate polyunsaturated fatty acids to the corresponding hydroperoxy derivatives even when these substrates are part of biomembranes and lipoprotein ester lipids.

## Introduction

Arachidonic acid lipoxygenases (ALOXs) catalyze the oxygenation of polyunsaturated fatty acids (PUFAs) to hydroperoxy derivatives^1–3^. In cells, these compounds are further metabolized to secondary products^4^ serving as signaling molecules^5^ in the pathogenesis of inflammatory^6^, hyperproliferative^7^, metabolic^8^ and neurological^9^ diseases. ALOX-isoforms frequently occur in highly developed plants^10^ and animals^11^, but they are less widely distributed in prokaryotes and lower eukaryotes^12^. In bacteria, they rarely occur^12,13^ and no functional ALOX genes have been detected so far in archaea^12^ and viruses^14^. In the human genome, six functional *ALOX* genes^11^ are present (*ALOX15, ALOX15B, ALOX12, ALOX12B, ALOX5, ALOXE3*) and the corresponding enzymes are expressed in different types of cells and tissues^1,2^. The mouse reference genome involves seven functional *Alox* genes. Here, an orthologous gene exists for each human ALOX isoform but in addition a functional *Aloxe12* gene is present, which constitutes a corrupted pseudogene in humans^11^.

Most mammalian ALOX isoforms prefer free PUFAs as substrate^15,16^. However, ALOX15 orthologs of different mammals^17–19^ are also capable of oxygenating esterified PUFAs even when these substrates are part of biomembranes^20–22^ or lipoproteins^23,24^. The membrane oxygenase activity of mammalian ALOX15 orthologs has been implicated in the maturational breakdown of mitochondria during erythrocyte development^25,26^ and recent experiments with *Alox15*^−/−^ mice confirmed this hypothesis^27^. The lipoprotein oxygenase activity of mammalian ALOX15 orthologs may play a role in atherogenesis^28–30^, and the following findings support this hypothesis: i) ALOX15 orthologs are capable of oxidizing low-density lipoproteins (LDL) to an atherogenic form^24,31^. ii) The enzyme is active in early atherosclerotic lesions, and specific ALOX15 products have been detected in the lesion lipids^32,33^. iii) *Alox15*^−/−^ mice were protected from aortic lipid deposition^34,35^. In addition, several ALOX-isoforms are capable of oxygenating PUFA-containing phospholipids when these substrates are offered as nano-disks^36,37^. When mouse Alox15b was used as a catalyst, the dominant formation of 15-HETE-containing ester lipids was observed. This finding was somewhat surprising since 8*S*-HETE is the major product of free arachidonic acid (AA) oxygenation^38^.

Although scattered literature reports suggested the capability of ALOX-isoforms to oxygenate PUFA containing phospholipids^18–20,36,39^ there is no systematic study, in which the oxygenase activity of different mammalian ALOX-isoform with complex substrates was directly compared. Moreover, there was no direct proof for the hypothesis that ALOX-catalyzed oxygenation of complex substrates does actually occur i*n vivo*. To address these points, we explored the activity of relevant mammalian ALOX-isoforms with different types of complex substrates, including different types of biomembranes and lipoproteins. We found that when normalized to their AA oxygenase activities, human and mouse ALOX15 orthologs exhibited high membrane-oxygenase activities. Mouse and human ALOX15B orthologs also catalyzed such reactions, but human ALOX12 was less effective.

## Results

### Functional characterization of mammalian ALOX isoforms

**Fig. 1:**
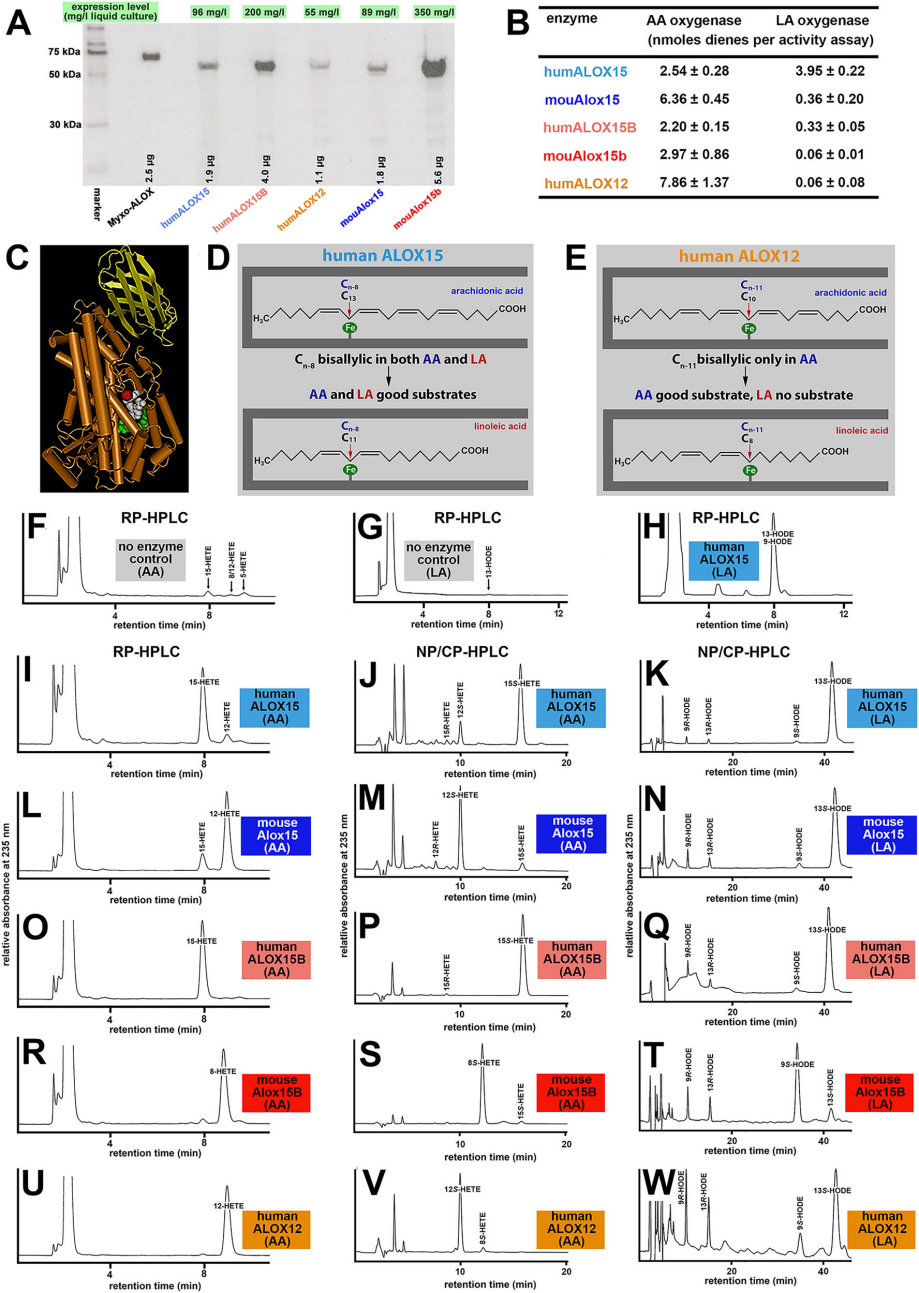
Expression and basic functional characterization of recombinant mammalian ALOX isoforms. The enzymes were expressed as N-terminal His-tag fusion proteins in Sf9 cells (human ALOX15) or E. coli (mouse Alox15, human ALOX15B, mouse Alox15b, human ALOX12). After cell lysis (sonication) aliquots of the lysate supernatants were used as enzyme source. **A** Quantitative Western blots. **B** Catalytic activities of ALOX-isoforms with both AA and LA as substrate (*n* = 4). **C** Strutural model of the rabbit ALOX15-AA-complex using the PDB coordinates 2P0M (yellow, N-terminal ß-barrel domain; brown, C-terminal catalytic domain; white, AA modeled into the substrate binding pocket; green, Triad amino acids formíng the bottom of the substrate binding pocket). **D** Schematic view of substrate (LA, AA) binding at the active site of mammalian ALOX15 orthologs. **E** Schematic view of substrate (LA, AA) binding at the active site of mamnmalian ALOX12. **F** RP-HPLC of the AA oxygenation formed during no-enzyme control incubations. **G** RP-HPLC of the LA oxygenation products formed during no-enzyme control incubations. **H** RP-HPLC of LA oxygenation products formed by recombinant human ALOX15. **I** RP-HPLC of the AA oxygenation products formed by human ALOX15. **J** NP/CP-HPLC of the AA oxygenation products formed by human ALOX15. **K** NP/CP-HPLC of the LA oxygenation products formed by human ALOX15. **L** RP-HPLC of the AA oxygenation products formed by mouse Alox15. **M** NP/CP-HPLC of the AA oxygenation products formed by mouse Alox15. **N** NP/CP-HPLC of the LA oxygenation products formed by mouse Alox15. **O** RP-HPLC of the AA oxygenation products formed by human ALOX15B. **P** NP/CP-HPLC of the AA oxygenation products formed by human ALOX15B. **Q** NP/CP-HPLC of the LA oxygenation products formed by human ALOX15B. **R** RP-HPLC of the AA oxygenation products formed by mouse Alox15b. **S** NP/CP-HPLC of the AA oxygenation products formed by mouse Alox15b. **T** NP/CP-HPLC of the LA oxygenation products formed by mouse Alox15b. **U** RP-HPLC of the AA oxygenation products formed by human ALOX12. **V** NP/CP-HPLC of the AA oxygenation products formed by human ALOX12. **W** NP/CP-HPLC of the LA oxygenation products formed by human ALOX12. Each activity assay (*n* = 4) was analyzed by RP-HPLC and  representative chromatograms are shown. For NP/CP-HPLC the conjugated dienes formed during the 3 min incubation period were prepared by RP-HPLC, pooled for each enzyme-substrate combinantion and analyzed once.

To characterize the oxygenase activity of mammalian ALOX-isoforms with complex substrates, we first expressed human ALOX15, mouse Alox15, human ALOX15B, mouse Alox15b and human ALOX12 as recombinant proteins. Since human ALOX15 was not well expressed in E. coli we expressed this enzyme in Sf9 insect cells. The enzymes were expressed at different levels (Fig. 1A) and in vitro activity assays (Fig. 1B) with both linoleic acid (LA) and arachidonic acid (AA) proved their functionality.

For ALOX-catalyzed oxygenation PUFAs are bound at the active site of the enzymes (Fig. 1C) and their alignment in relation to the non-heme iron is important for the reactivity. For human ALOX15 AA is oriented at the active site in such a way that the bisallylic methylene C13 (n-8^th^ carbon) is localized in close proximity to the iron (Fig. 1D, upper panel). In LA the n-8^th^ carbon is also bisallylic (Fig. 1D, lower panel) and thus, LA is also a good substrate for this enzyme. For human ALOX12 AA is bound in a different way (Fig. 1E, upper panel). Here the n-11^th^ carbon is localized close to the iron, which leads to AA C12 oxygenation. However, in LA the n-11^th^ carbon is not bisallylic (Fig. 1E, lower panel) and thus, the LA oxygenase activity of this enzyme should be  compromised.

Analyzing the PUFA oxygenation products formed during the activity assays we found that only small amounts were detected in no-enzyme control incubation (Fig. 1F+G) and in other control samples (Fig. S1). Human ALOX15 produced 13-HODE from LA (Fig. 1H) but AA (Fig. 1I) was oxidized to a 10 : 1 mixture of 15-HETE and 12-HETE. Consistent with previous data^18^ for all products the S-enantiomers were dominant (Fig. 1J+K). For mouse Alox15^40^, an inverse product mixture (15-HETE / 12-HETE ratio of about 2 : 10) was analyzed (Fig. 1L, M). For LA oxygenation 13S-HODE was the major product (Fig. 1N) and in corresponding control incubations products were absent (Fig. S2). For human ALOX15B, 15S-HETE was the exclusive AA oxygenation product (Fig. 1O+P) and 13S-HODE was formed from LA (Fig. 1Q). Mouse Alox15b oxygenated AA mainly to 8S-HETE (Fig. 1R+S) confirming previous results with this enzyme^38,41,42^. The major LA oxygenation product was 9S-HODE but smaller amounts of 13S-HODE were also detected (Fig. 1T). Here again, no product formation was seen in control incubations (Fig. S3+4). As expected^43,44^, 12S-HETE was the major AA oxygenation product of human ALOX12 (Fig. 1U+V) and with LA (Fig. 1W) 13S-HODE was dominant. In different control incubations no product formation was seen (Fig. S5). For pure recombinant human ALOX15 a 10: 1 mixture of 15-HETE and 12-HETE (Fig. S6) was analyzed as major AA oxygenation products and this data is consistent with the results obtained with the crude enzyme preparation (Fig. 1I).

### Most ALOX isoforms exhibit a liposome oxygenase activity

**Fig. 2:**
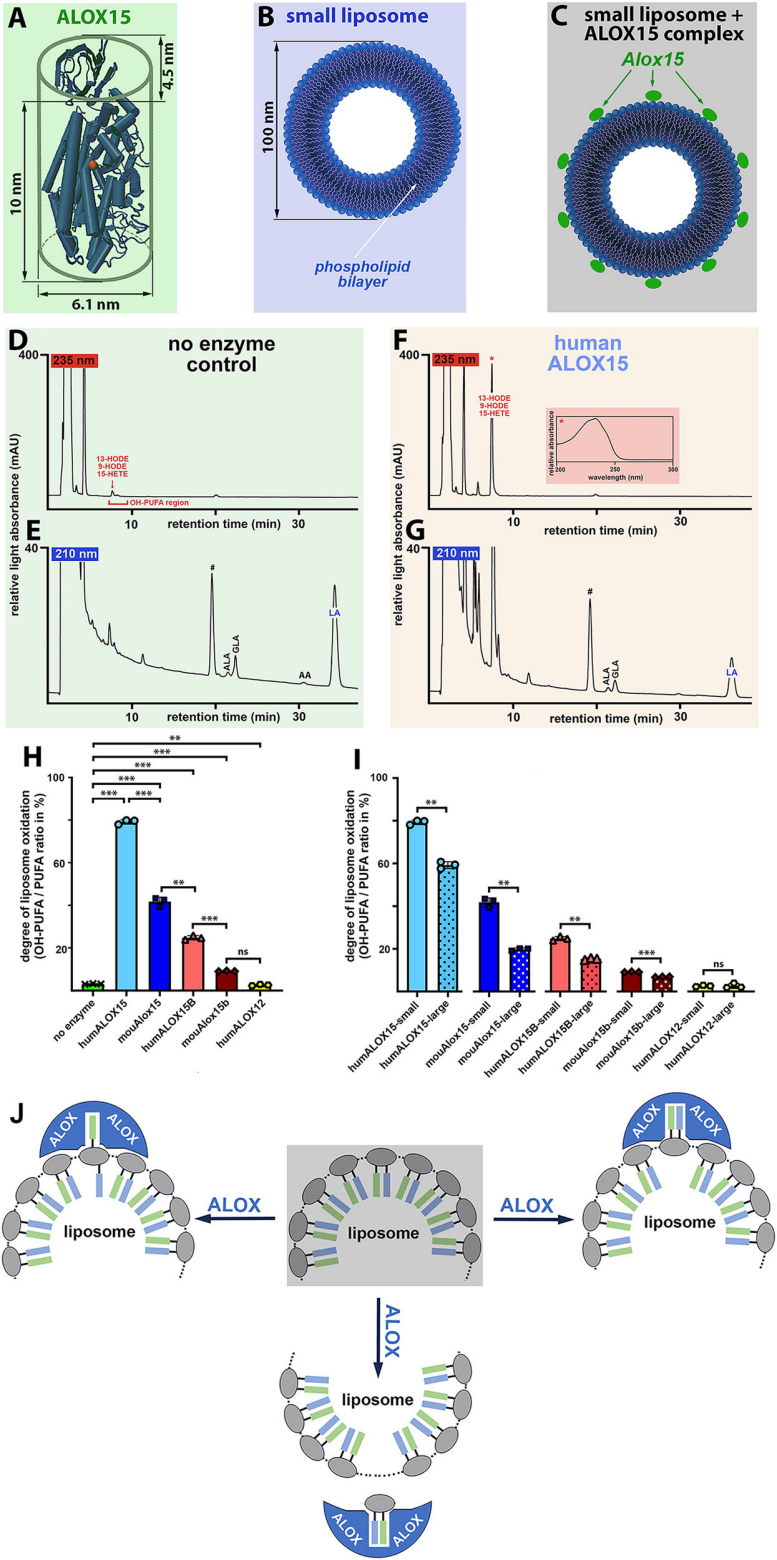
Liposome oxygenase activity of mammalian ALOX isoforms. **A** Schematic view of the 3D structure of rabbit ALOX15 serving as model for the human ortholog. The red circle represents the catalytic non-heme iron. **B** Schematic structure of small liposomes. **C** ALOX15 binding at the surface of liposomes (size relation). **D** RP-HPLC analysis of hydrolyzed lipid extracts of no enzyme control incubations at 235 nm (oxygenated PUFAs). **E** RP-HPLC analysis of hydrolyzed lipid extracts of no enzyme control incubations at 210 nm (non-oxygenated PUFAs). **F** RP-HPLC analysis of hydrolyzed lipid extracts of hALOX15 incubations at 235 nm (oxygenated PUFAs). **G** RP-HPLC analysis of hydrolyzed lipid extracts of hALOX15 incubations at 210 nm (non-oxygenated PUFAs). **H** Small liposome oxygenase activity of different ALOX-isoforms; (*n* = 3 for each liposome enzyme combination). **I** Comparison of small and large liposome oxygenase activities of different ALOX-isoforms; *n* = 3 for each liposome enzyme combination). **J** Schematic representation of ALOX15 binding at the surface of liposomes. ^#^ unidentified compound. **, *p* < 0.01; *** *p* < 0.0001; ns not significant, ALA alpha linoleic acid, GLA gamma linoleic acid, AA arachidonic acid, LA linoleic acid. An oxidation degree of 80% indicated that 8 out of 10 polyenoic fatty acids are present as oxygenated derivatives.

The crystal structure of rabbit ALOX15 resembles an elliptic cylinder **(**Fig. 2A**)**. Its active site is an internal cavity with connection to the protein surface^45^ and the deepness of the substrate binding pocket is limited by the Triad amino acids (green in Fig. 1C). Free AA (white in Fig. 1C) fits into this substrate binding pocket and is bound in proximity to the non-heme iron (red spheres in Fig. 1C+2A). Liposomes^46^ are far too big (Fig. 2B) to enter the active site (Fig. 2C). Nevertheless, human ALOX15 oxygenates liposome phospholipids (Fig. 2D–I). RP-HPLC analysis of hydrolyzed lipid extracts of no-enzyme control incubations (Fig. 2D) revealed only small amounts of oxygenated PUFAs and similar data were obtained for other control incubations (Fig. S7–S12). In contrast, non-oxygenated PUFAs were detected in large amounts and LA and gamma linolenic acid (GLA) were dominant (Fig. 2E). However, when we analyzed the extracts of human ALOX15 treated liposomes (Fig. 2D*vs*. 2F) large amounts of oxygenated PUFAs were observed and we found an anti-parallel reduction of LA levels (Fig. 2E*vs*. 2G).

Next, we performed similar incubations with the other ALOX isoforms. When normalized to identical AA oxygenase activities human ALOX15 exhibited the highest liposome oxygenase activity followed by mouse Alox15, human ALOX15B and mouse Alox15b (Fig. 2H). Only small amounts of conjugated dienes were detected in the non-hydrolyzed lipid extracts (Fig. S7–S12) indicating that esterified LA was the major substrate. For human ALOX12 the OH-PUFA / PUFA ratio was lower than that of the no-enzyme control incubations and thus, this enzyme does not exhibit a liposome oxygenase activity. Additional experiments with liposomes of a different size indicated (Fig. 2I) that small liposomes (0.1 µm) were better substrates than bigger ones (1 µm).

Since liposomes are large particles (Fig. 2C) it remained unclear how such big substrates can be oxidized by ALOX isoforms. There are several ways to explain our findings and some of them are visualized in Fig. 2J. Since penetration of the enzymes into the phospholipid bilayer is unlikely, the ALOX-isoforms should bind at the liposome surface. The substrate binding pocket of the enzyme may offer a hydrophobic environment so that either one or the two fatty acids of a phospholipid molecule may penetrate into the active site (upper panel of Fig. 2J). However, the most probable mechanism would be that the enzyme “extracts” a complete phospholipid molecule form the lipid bilayer (lower panel of Fig. 2J), aligns it at the active site and catalyzes specific oxygenation of the PUFA moiety. The oxidized phospholipid may subsequently be re-integrated into the lipid bilayer of the liposome.

### Esterified 13S-HODE was the major oxygenation product formed from liposomes

**Fig. 3:**
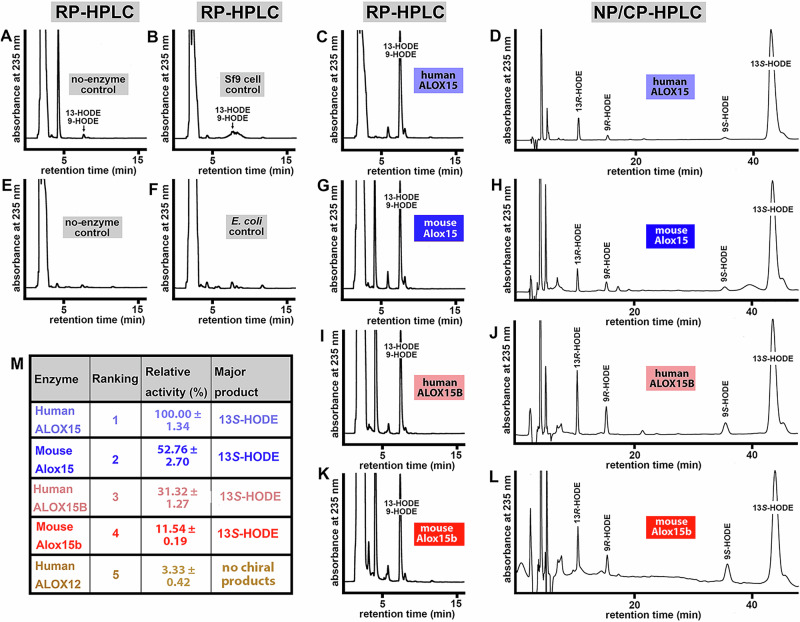
Structural identification of liposome oxygenation products formed by different ALOX-isoforms. **A** RP-HPLC analysis of no-enzyme (PBS) control incubation; *n* = 3 and a representative chromatogram is shown. **B** RP-HPLC analysis of incubation samples, in which the cellular lysate supernatant of Sf9 cells were used, which were infected with non-recombinant baculovirus; *n* = 3 and a representative chromatogram is shown. **C** RP-HPLC analysis of hALOX15 incubation samples; *n* = 3 and a representative chromatogram is shown. **D** NP/CP-HPLC analysis of the hALOX15 incubation samples. **E** RP-HPLC analysis of no-enzyme (PBS) control incubation samples; *n* = 3 and a representative chromatogram is shown. **F** RP-HPLC analysis of incubation samples, in which the cellular lysate supernatant of E. coli cells was used, which were transformed with a non-recombinant (empty) expression plasmid; *n* = 3 and a representative chromatogram is shown. **G** RP-HPLC analysis of mAlox15 incubation samples; *n* = 3 and a representative chromatogram is shown. **H** NP/CP-HPLC analysis of mAlox15 incubation samples. **I** RP-HPLC analysis of hALOX15B incubation samples; *n* = 3 and a representative chromatogram is shown. **J** NP/CP-HPLC analysis of hALOX15B incubation samples. **K** RP-HPLC analysis of mAlox15b incubation samples; *n* = 3 and a representative chromatogram is shown. **L** NP/CP-HPLC analysis of mAlox15b incubation samples. **M** Relative liposome oxygenase activities of different ALOX isoforms. Human ALOX15 was et 100% and the major reaction product are specified, *n* = 3 for each enzyme. For NP/CP-HPLC the conjugated dienes of the 3 incubation samples were prepared by RP-HPLC, pooled and analyzed once.

Since LA was the dominant PUFA in our liposome preparations esterified 13-HODE and/or 9-HODE should be the major oxygenation products. No conjugated dienes were detected in two different control incubations (Fig. 3A, B). In contrast, human ALOX15 produced large amounts of conjugated dienes (Fig. 3C) and 13*S*-HODE was dominant (Fig. 3D). No OH-PUFAs were detected in two different *E. coli* control incubations (Fig. 3E, F) but the major product of mouse Alox15 catalyzed LA oxygenation was 13*S*-HODE (Fig. 3G, H). Human ALOX15B did also produce 13*S*-HODE (Fig. 3I, J) and this result was expected from the reaction specificities of the enzymes with free LA (Fig. 1Q). Although the liposome oxygenase activity of mouse Alox15b was rather small (Fig. 2H) the product pattern was highly specific. Here again, 13*S*-HODE was identified as major oxygenation product (Fig. 3K, L). This finding was somewhat surprising since mouse Alox15b oxygenated free LA dominantly to 9*S*-HODE (Fig. 1T). The possible reasons for this difference have been discussed in a previous study^36^ and may be related to an inverse head-to-tail substrate alignment at the active site. Since human ALOX12 did not exhibit a liposome oxygenase activity (Fig. 2H, I), non-chiral LA-derived autoxidation products were detected for this enzyme. A ranking of the liposome oxygenase activities of the different ALOX isoforms is given in Fig. 3M.

### Substrate docking studies and molecular dynamic simulations

**Fig. 4:**
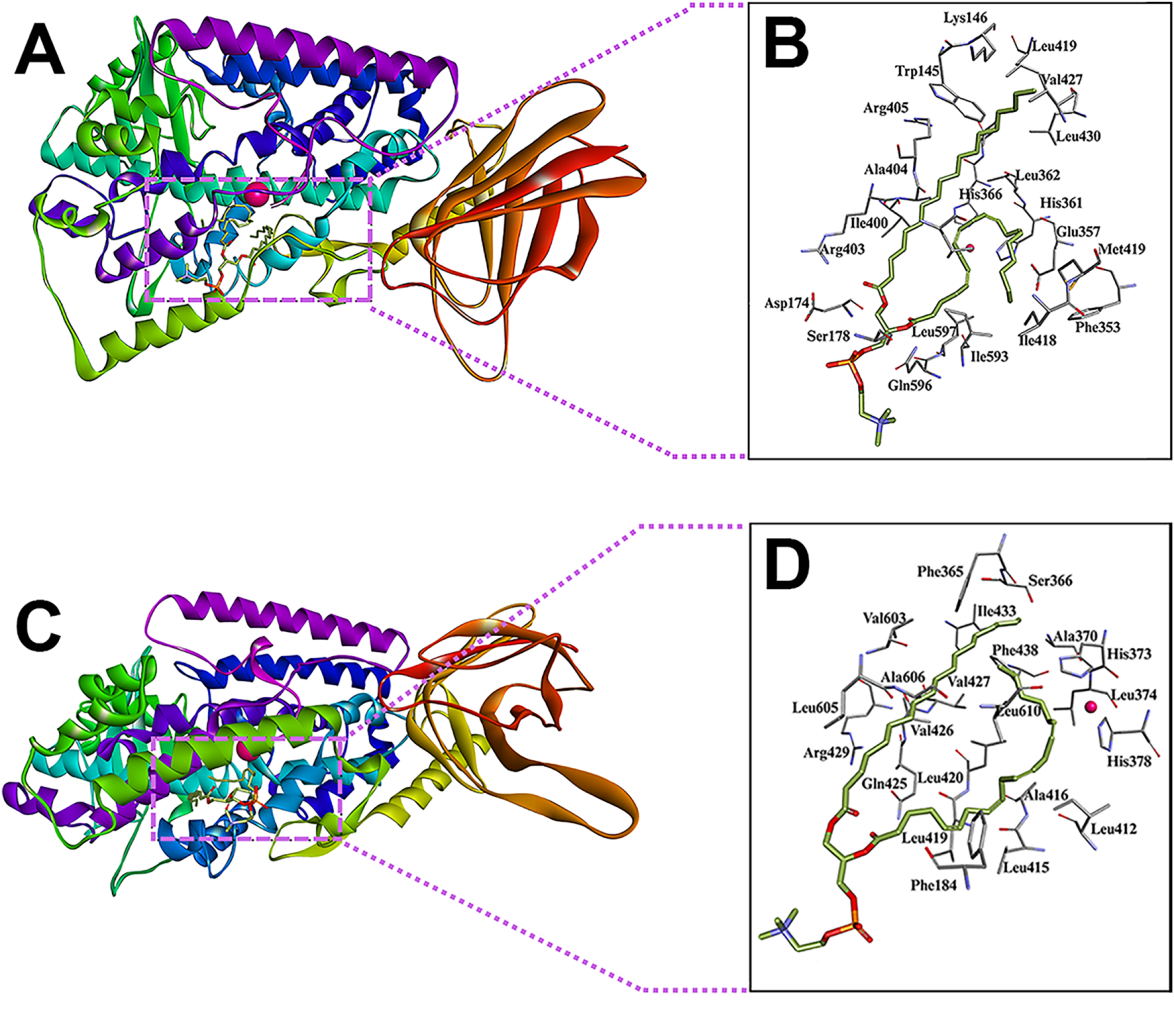
Molecular docking studies of a phospholipid molecule (SAPC) at the active side of rabbit ALOX15 and human ALOX15B. Molecular docking studies were carried out as described in the Material and Method section. **A** rALOX15-SAPC complex. **B** Amino acids stabilizing the rALOX15-SAPC complex. **C** hALOX15B-SAPC complex. **D** Amino acids stabilizing the hALOX15B-SAPC complex. In the selected docking pose of SAPC in human ALOX15B, the polar head group of the substrate was not positioned in close proximity to any active site amino acid, and thus, we did not show such an interaction. However, during the MD simulation, the polar head group moved around and was finally stabilized by interacting with the side-chains of Arg429 and Gln425.

When compared with PUFAs, phospholipids are bulky molecules, and it has never been explored whether the complete phospholipid molecule fits into the substrate binding pocket of mammalian lipoxygenases. To address this question, we first performed molecular docking studies of 1-stearoyl-2-arachidonoyl-sn-glycero-3-phosphocholine (SAPC) at the active site of rabbit ALOX15 and human ALOX15B. For such in silico dockings the 3D-structures for the enzymes must be available but this is not the case for human ALOX15 and the different mouse ALOX isoforms used in our functional studies (Fig. 2). However, rabbit ALOX15 shares a high degree of amino acid identity (some 86%) with both human and mouse ALOX15 orthologs and thus, the 3D structure of the rabbit enzyme may serve as structural model for the enzyme orthologs of mice and humans. Exploring rabbit ALOX15-SAPC interaction, we found three plausible docking poses (Fig. S13) for the ligand and the binding energies of the three enzyme-substrate complexes were very similar. For further investigations, we selected pose 3, in which the polar head group of the SAPC molecule was oriented towards a solvent-exposed region of the substrate binding pocket, while the hydrophobic acyl chains were localized in proximity of the non-heme iron (Fig. 4A, B). The methyl terminus of the AA chain contacted the Triad determinants (Phe353, Ile418+Met419, Ile593), which play a role for the reaction specificity of mammalian ALOX15 orthologs^47,48^. The saturated sn-1 acyl chain extended deeply into the hydrophobic pocket, where it was stabilized by interactions with the side chains of Trp145, Leu149, His361, Leu362, Leu367, and Leu430 (Fig. 4B). The polar phospholipid head group was positioned near the side chains of Asp174, Ser178, Gln596 and Ser178 forming a stabilizing hydrogen bond network (Fig. 4B). However, despite these favorable interactions, several steric clashes were also observed. The bulky sn-2 chain unfavorably contacts Ile400, Ala404 and Val427 and the polar head group interacts repulsively with the side chain of Asp174 (Fig. 4B).

For the human ALOX15B-SAPC-complex the AA moiety did also adopted a U-shaped conformation and C13 of the hydrocarbon chain remained in close proximity to the iron (Fig. 4C, D). The complex was stabilized by interactions of the AA chain with the iron-coordinating His373 and His378, but also with the side chains of Leu374, Ile412, Leu415, Ala416, Leu419, and Leu420 (amino acid numbering according to the PDB entry 4NRE). The sn-1 stearoyl chain was also buried inside the substrate binding pocket, and its position was stabilized by hydrophobic interactions with the side chains of Leu605, Val603, Phe365, Ile433, and Phe438 (Fig. 4C). Here again, some steric clashes were observed. The side chains of Ser366, Val426 and Ala606 are partly displaced by the substrate, and this displacement restricts the conformational flexibility of the ligand within the substrate binding pocket.

To test the stability of the enzyme-substrate complexes, molecular dynamics (MD) simulations were performed. For the rabbit ALOX15-SAPC complex (supplemental movie M1) the AA acyl chain retained its U-shaped structure throughout the 100 ns simulation period and its terminal methyl group remained in contact with the Triad determinants (Phe353, Ile418+Met419, Ile593). Interestingly, during the simulation period, the choline moiety of SAPC underwent a conformational flip and established a stable hydrogen bond with the side chain of Asp174. The phosphate group of the substrate remained firmly engaged, maintaining its hydrogen bond with Gln596. In addition, the carbonyl oxygen of the stearoyl chain formed a stable hydrogen bond with Ser178, stabilizing the enzyme-substrate complex.

In the human ALOX15B-SAPC-complex the AA chain did also preserved its U-shaped conformation during the entire simulation period and the side chains of Phe184, Leu415, Ala416, and Leu420 stabilized this structure (supplemental movie M2). The stearoyl chain remained associated with a cluster of hydrophobic amino acids (Phe365, Phe437, Ala606, Leu609, Leu610). Interestingly, the trimethylamine group of the polar head group approached the aromatic ring of Tyr185 to establish a stabilizing π-cation interaction. Moreover, the substrates’ phosphate group is part of a water-mediated hydrogen bond network, which also includes the side chains of Gln425 and Arg429.

### Reactivity of ALOX-isoforms with mitochondrial membranes

**Fig. 5:**
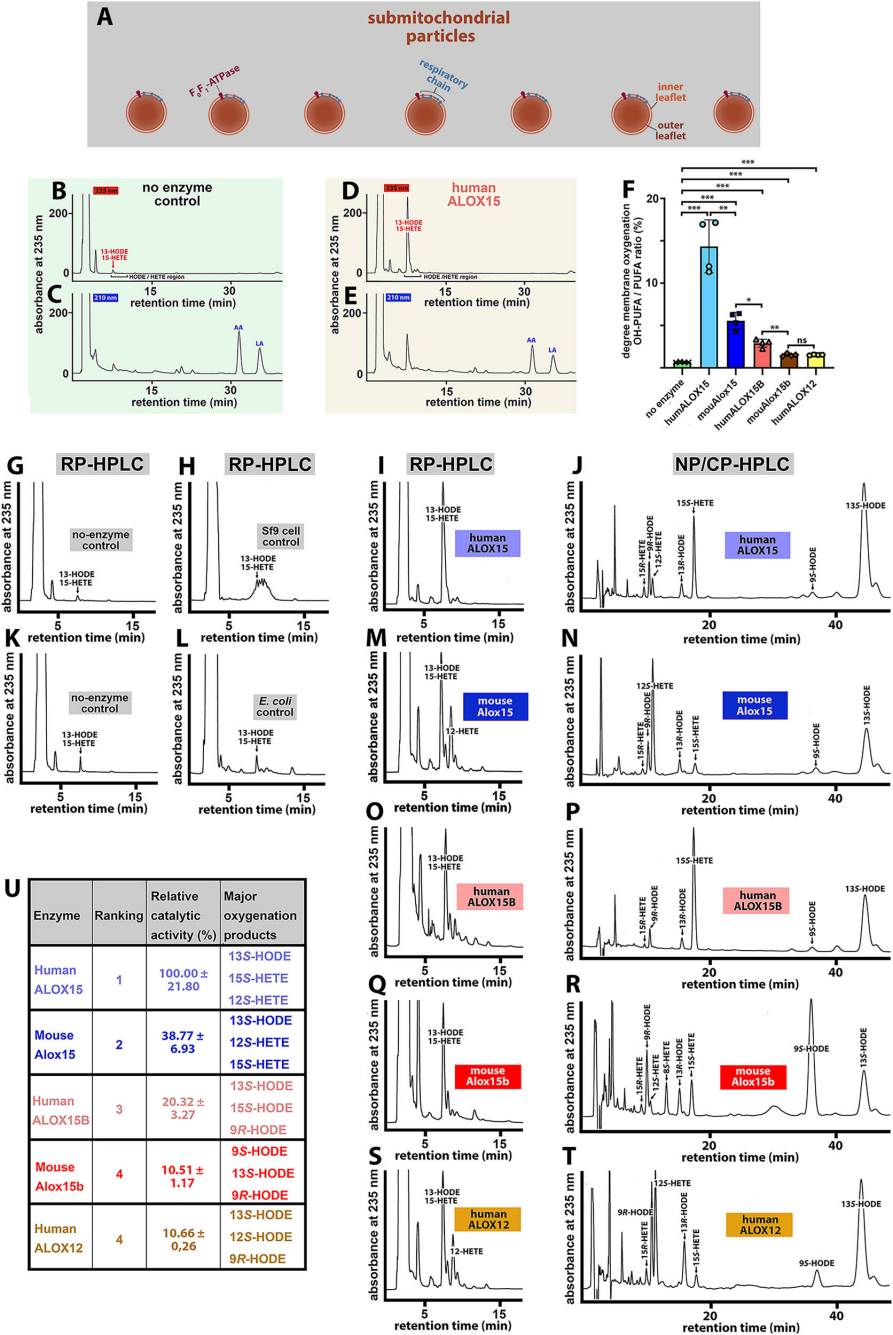
Mitochondrial membrane oxygenase activities of mammalian ALOX-isoforms. **A** Schematic representation and structure of submitochondrial particles used as substrate for determination of the mitochondrial membrane oxygenase activity of the different ALOX isoforms. **B**,**C** RP-HPLC analysis of no-enzyme (PBS) control incubation samples. **D**,**E** RP-HPLC analysis of hALOX15 incubation samples. **F** OH-PUFA / PUFA ratios calculated as suitable measure for the degree of oxidation of the membrane lipids (*n* = 4, *, *p* < 0.05; **, *p* < 0.01, *** *p* < 0.0001; ns, not significant. **G** RP-HPLC analysis of no-enzyme (PBS) control incubation. **H** RP-HPLC analysis of incubation samples, in which the cellular lysate supernatant of Sf9 cells were used, which were infected with non-recombinant baculovirus. **I** RP-HPLC analysis of hALOX15 incubation samples. **J** NP/CP-HPLC analysis of hALOX15 incubation samples. **K** RP-HPLC analysis of no-enzyme (PBS) control incubation samples. **L** RP-HPLC analysis of incubation samples, in which the cellular lysate supernatant of E. coli cells was used, which were transformed with a non-recombinant (empty) expression plasmid. **M** RP-HPLC analysis of mAlox15 incubation samples. **N** NP/CP-HPLC analysis of mAlox15 incubation samples. **O** RP-HPLC analysis of hALOX15B incubation samples. **P** NP/CP-HPLC analysis of hALOX15B incubation samples. **Q** RP-HPLC analysis of mAlox15b incubation samples. **R** NP/CP-HPLC analysis of mAlox15b incubation samples. **S** RP-HPLC analysis of hALOX12 incubation samples. **T** NP/CP-HPLC analysis of hALOX12 incubation samples. **U** Relative liposome oxygenase activities of different ALOX isoforms. hALOX15 activity was set to 100% and the major reaction products are specified.

Mitochondrial membranes are suitable substrates for rabbit and pig ALOX15 orthologs^18–20,37^. ALOX15 dependent membrane oxygenation has recently been implicated in ferroptotic signaling but for this activity the phosphatidyl ethanolamine binding protein 1 (PEBP1) was required^49^. To explore whether other ALOX isoforms also exhibit a mitochondrial membrane oxygenase activity we incubated electron transfer particles (Fig. 5A), which constitute functional inside-out vesicles of beef heart inner mitochondrial membranes^50^, with recombinant ALOX preparations. In no-enzyme control incubations we did not detect the formation of specific ALOX15 products (Fig. 5B) but we observed large amounts of non-oxygenated PUFAs (Fig. 5C). Although the AA peak was somewhat higher than the LA peak calibration of the HPLC scale indicated that LA was the dominant PUFA in these types of membranes. In fact, at 210 nm AA exhibits a 4-fold higher molar extinction coefficient than LA and thus, similar peak areas do not indicate similar abundance. When the membranes were incubated for 15 min with human ALOX15 large amounts of oxygenation products were observed (Fig. 5B*vs*. 5D) and these products were absent in different control incubations (Fig. S14). Moreover, the PUFA content was reduced during the incubation period (Fig. 5C*vs*. 5E). When we calculated the OH-PUFA / PUFA ratio for human ALOX15 (Fig. 5F) we found that after a 15 min incubation period about 15% of the PUFAs in the mitochondrial membrane lipids were present as oxygenated derivatives. In no enzyme control incubations this ratio was only about 0.3% (Fig. 5F).

Mitochondrial membranes carry both, AA and LA as major PUFAs (Fig. 5C, E) and thus, HETE and HODE isomers were expected as major oxygenation products. As indicated in Fig. 5G, H,K, L OH-PUFAs were hardly detected in control incubations. For human ALOX15, 13S-HODE was the major oxygenation product and smaller amounts of 15S-HETE were also observed (Fig. 5I, J). For mouse Alox15 (Fig. 5M, N), 13S-HODE and 12S-HETE were identified as major oxygenation products. Human ALOX15B exhibits a relatively small (20% of human ALOX15) mitochondrial membrane oxygenase activity (Fig. 5F) but the enzyme produced a highly specific product pattern (Fig. 5O, P). 13S-HODE (LA oxygenation) and 15S-HETE (AA oxygenation) were detected in similar quantities although LA was more abundantly present in these types of membranes (Fig. 5C, E). These data suggest that human ALOX15B prefers AA over LA when the substrates are presented as ester lipids and this finding is consistent with our results of the free PUFA oxygenase activity of this enzyme (Fig. 1B). Mouse Alox15b exhibited an even lower mitochondrial membrane oxygenase activity than the human enzyme ortholog (Fig. 5F), but for this enzyme we observed a surprising product pattern. Free AA is almost exclusively converted to 8S-HETE (Fig. 1R, S) but this compound was only a minor side product when mitochondrial membranes were used as substrate. As major products LA-derived 9S- and 13S-HODE were identified (Fig. 5Q, R). The corresponding R-enantiomers were present in much smaller quantities and this was also the case for 12S-HETE, 15S-HETE. In a previous report^36^ it has been shown that mAlox15b converted AA-containing phospholipids presented in form of nanodiscs are mainly oxidized to 15S-HETE containing phospholipids and this was not the case for mitochondrial membranes. Human ALOX12 exhibited only a low mitochondrial membrane oxygenase activity (Fig. 5F) and the pattern of oxygenation products was rather unspecific (Fig. 5S, T). With mitochondrial membranes the following enzyme ranking was calculated (Fig. 5U): human ALOX15 > mouse Alox15 > human ALOX15B > mouse Alox15b = human ALOX12.

### Reactivity of ALOX-isoforms with endoplasmic membranes

**Fig. 6:**
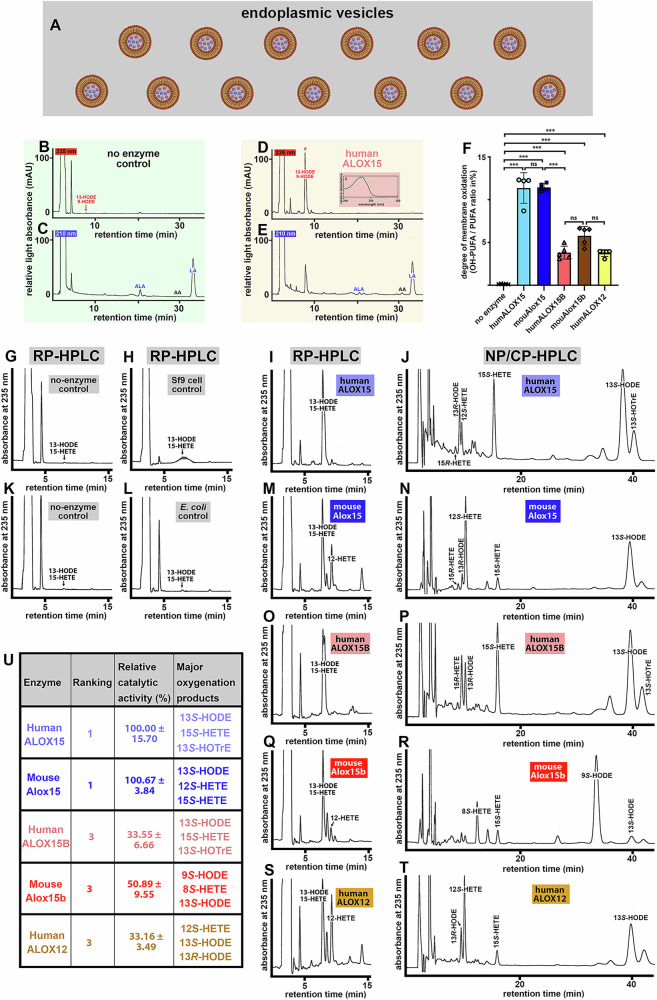
Endoplasmic membrane oxygenase activities of mammalian ALOX-isoforms. **A** Schematic representation and structure of endoplasmic membrane vesicles used as substrate for determination of the endoplasmic membrane oxygenase activity of the different ALOX isoforms. **B**,**C** RP-HPLC analysis of no-enzyme (PBS) control incubation samples. **D**,**E** RP-HPLC analysis of hALOX15 incubation samples. **F** OH-PUFA / PUFA ratios calculated as suitable measure for the degree of oxidation of the membrane lipids (*n* = 4-5, *, *p* < 0.05; **, *p* < 0.01, *** *p* < 0.0001; ns, not significant. **G** RP-HPLC analysis of no-enzyme (PBS) control incubation. **H** RP-HPLC analysis of incubation samples, in which the cellular lysate supernatant of Sf9 cells were used, which were infected with non-recombinant baculovirus. **I** RP-HPLC analysis of hALOX15 incubation samples. **J** NP/CP-HPLC analysis of h ALOX15 incubation samples. **K** RP-HPLC analysis of no-enzyme (PBS) control incubation samples. **L** RP-HPLC analysis of incubation samples, in which the cellular lysate supernatant of E. coli cells was used, which were transformed with a non-recombinant (empty) expression plasmid. **M** RP-HPLC analysis of mAlox15 incubation samples. **N** NP/CP-HPLC analysis of mAlox15 incubation samples. **O** RP-HPLC analysis of hALOX15B incubation samples. **P** NP/CP-HPLC analysis of hALOX15B incubation samples. **Q** RP-HPLC analysis of mAlox15b incubation samples. **R** NP/CP-HPLC analysis of mAlox15b incubation samples. **S** RP-HPLC analysis of hALOX12 incubation samples. **T** NP/CP-HPLC analysis of hALOX12 incubation samples. **U** Relative endoplasmic membrane oxygenase activities of different ALOX isoforms. hALOX15 activity was set to 100% and the major reaction products are specified.

Endoplasmic vesicles (Fig. 6A) are differently structured than mitochondrial membranes but they also constitute a substrate for rabbit ALOX15^21^. Unfortunately, the pattern of reaction products has not been explored and for other ALOX-isoforms corresponding experiments have not been performed. Analyzing the hydrolyzed lipid extracts of no-enzyme (PBS) control incubations we did not find significant amounts of conjugated dienes (Fig. 6B) and LA was the dominant PUFA in this type of biomembranes (Fig. 6C). Smaller amounts of ALA were also present but AA was virtually absent (Fig. 6C). When the endoplasmic membranes were incubated with human ALOX15 large amounts of oxygenation products were detected in RP-HPLC (Fig. 6D). Following the chromatogram at 210 nm we still detected non-oxidized LA (Fig. 6E) and this data indicates that only a small share of LA present in the membranes was oxygenated during the incubation period. When we compared the OH-PUFA / PUFA ratios we found that human ALOX15 and mouse Alox15 exhibited similar endoplasmic membrane oxygenase activities. Although the corresponding activities of the other ALOX-isoforms were lower human ALOX15B, mouse Alox15b and human ALOX12 also exhibited ER membrane oxygenase activities (Fig. 6F).

Since LA was the dominant PUFA in our endoplasmic membrane preparations (Fig. 6C, E), we expected 13-HODE and/or 9-HODE as major oxygenation products. No conjugated dienes were detected in the control incubations (Fig. 6G, H, K, L). For human ALOX15 13S-HODE was identified as major product (Fig. 6I, J). Surprisingly, significant amounts of 15S-HETE and 13S-HOTrE were also detected (Fig. 6J) although the parent PUFAs were only present low levels (Fig. 6C, E). For mouse Alox15 (Fig. 6M, N) 12S-HETE and 13S-HODE were identified as major oxygenation products. For human ALOX15B, we predicted 15S-HETE and 13S-HODE as major oxygenation products and this assumption was confirmed experimentally (Fig. 6O, P). For mouse Alox15b chiral 9*S*-HODE was the major oxygenation product and 8*S*-HETE was detected in smaller amounts (Fig. 6Q, R). Thus, for this enzyme the product pattern of ER membrane oxygenation was similar to that of mitochondrial membrane oxidation (Fig. 5Q, R) but different from that of liposome oxygenation (Fig. 4K, L). When incubated with ER membranes human ALOX12 mainly produced 12S-HETE and 13S-HODE but smaller amounts of 15S-HETE were also detected (Fig. 6S, T). The following enzyme ranking was observed for ER membrane oxygenation (Fig. 6U): human ALOX15 = mouse Alox15 > human ALOX15B = mouse Alox15b = human ALOX12.

### Lacking reactivity of ALOX-isoforms with erythrocyte plasma membranes

To test whether our ALOX-isoforms exhibit a plasma membrane oxygenase activity we prepared human erythrocyte ghosts^51^ and incubated them with our recombinant enzyme preparations. We found (Table S1) that neither of our enzyme preparations was capable of oxidizing the membrane ester lipids. Similar conclusions were drawn from previous  in vitro hemolysis assays^52^. The molecular basis for the lacking erythrocyte ghost oxygenase activity of the recombinant ALOX isoforms have not been studied but a lack of PUFAs was excluded (Fig. S15).

### Reactivity of ALOX-isoforms with lipoproteins

**Fig. 7:**
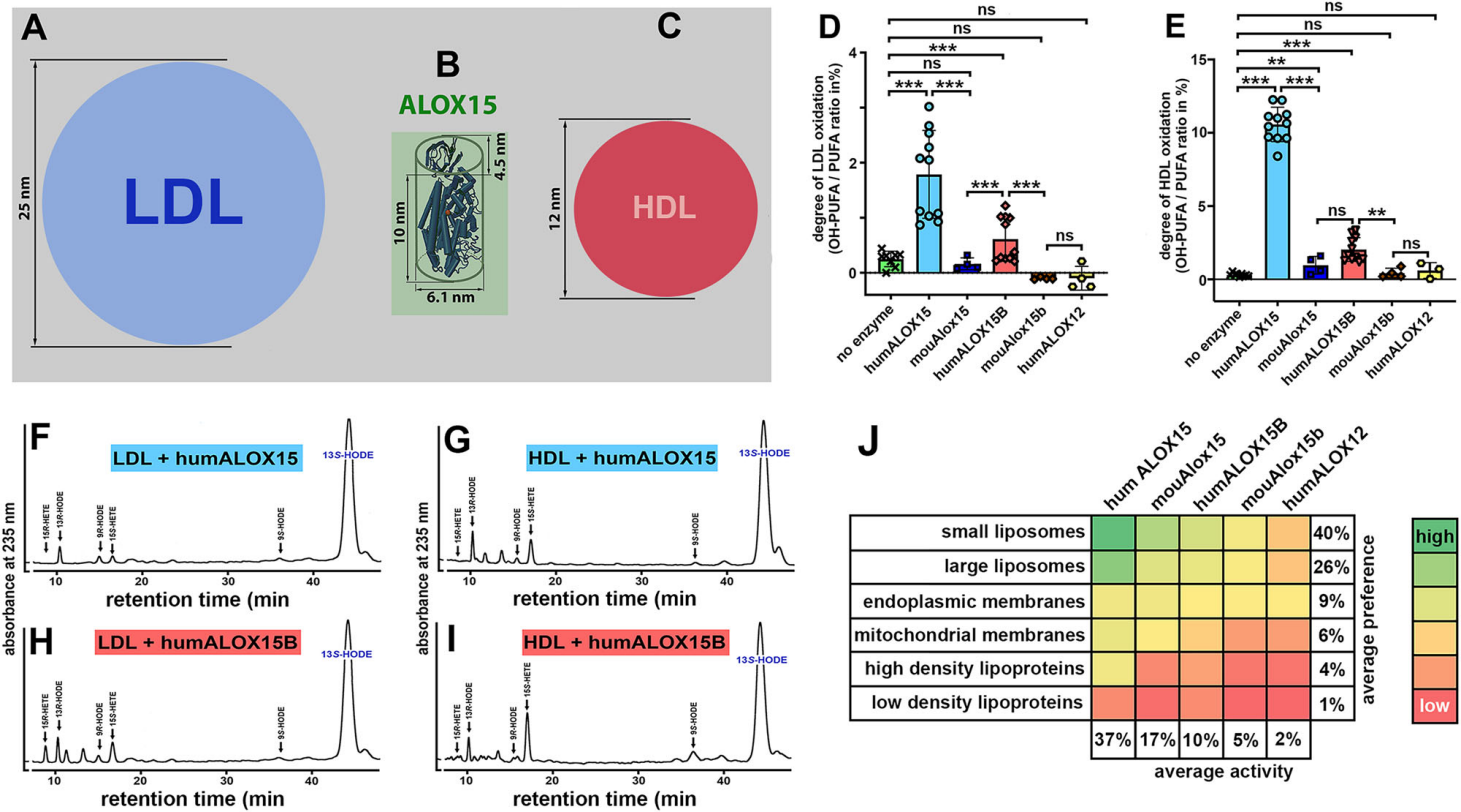
Lipoprotein oxygenase activity of mammalian ALOX isoforms. **A-C** Schematic representation and size comparison of rabbit ALOX15 and human lipoproteins (LDL blue, HDL red). **D** LDL oxygenase activities of different ALOX isoforms (*n* = 4–11; **, *p* < 0.01; ***, *p* < 0.001, ****, *p* < 0.0001; ns, not significant). **E** HDL oxygenase activities of different ALOX isoforms (*n* = 3–11; **, *p* < 0.01; ***, *p* < 0.001, ****, *p* < 0.0001; ns, not significant). **F** NP/CP-HPLC analysis of products formed from LDL by hALOX15. **G** NP/CP-HPLC analysis of products formed from HDL by hALOX15. **H** NP/CP-HPLC analysis of products formed from LDL by hALOX15B. **I** NP/CP-HPLC analysis of products formed from HDL by hALOX15B. **J** Heatmap summarizing the capability of different ALOX isoforms to oxygenate different PUFA-containing lipid-protein assemblies.

Lipoproteins (Fig. 7A) function as lipid-carrier proteins in the blood^53^ and lipoprotein oxidation has been implicated in atherogenesis^28,30^. Rabbit and pig ALOX15 orthologs are capable of oxidizing LDL and HDL^24,54,55^ but for other mammalian ALOX-isoforms corresponding data have not been published. Human lipoproteins are bigger than mammalian ALOX isoforms (Fig. 7A–C) and thus, they cannot bind at the active site of the enzymes. On the other hand, they carry large amounts of PUFAs (Fig. S16) and thus, lipoproteins constitute potential ALOX substrates. To evaluate the lipoprotein oxygenase activity of different ALOX isoforms, we incubated freshly prepared human LDL and HDL with our enzyme preparations. Analyzing the hydrolyzed lipid extracts of no-enzyme control incubations we quantified OH-PUFAs / PUFA ratio of about 0.25% (Fig. 7D). When human ALOX15 and human ALOX15B (Fig. 7D) were included in the incubation samples OH-PUFA /PUFA ratios of 1.8% and 0.6%, respectively, were determined. For mouse Alox15, mouse Alox15b and human ALOX12 the OH-PUFA / PUFA ratios were not significantly different from that of the control incubations (Fig. 7D). With HDL similar results were obtained (Fig. 7E). We also analyzed the pattern of the oxygenation products and found that 13*S*-HODE was the major oxygenation product of human ALOX15 and human ALOX15B-catalyzed LDL- and HDL-oxygenation (Fig. 7F–I).

To summarize the substrate preferences of the tested enzymes and to rank the ALOX isoforms with respect to their oxygenase activities for the different substrates, we constructed a heatmap (Fig. 7J). This data indicates that human ALOX15 exhibited the highest catalytic activity with most substrates, followed by mouse Alox15 and human ALOX15B. Among the different substrates, small liposomes were preferred by all ALOX-isoenzymes. In contrast, human LDL and HDL were less effective substrates.

### In vivo activity of ALOX15 in genetically modified mice

Erythrocytes are devoid of ALOX15, but other blood cells express the enzyme^56^. Since blood cells exchange lipids with the blood plasma, there is the possibility that PUFA-containing phospholipids, which have been oxygenated by intracellular ALOX15, might be present in the plasma lipids. To test this hypothesis, we profiled the plasma oxylipidomes of mice in which the *Alox15* gene was modified. For this purpose, we extracted the blood plasma lipids and analyzed the oxylipidomes of the hydrolyzed and non-hydrolyzed extracts by LC-MS/MS. For all animals, we observed that only small amounts of oxygenated PUFAs were present in the non-hydrolyzed lipid extracts. In contrast, much larger quantities of these products were identified in the hydrolyzed extracts, and this data indicates that the vast majority of the oxidized PUFAs are present in the blood plasma ester lipids. However, this data does not prove that intracellular ALOX15 oxygenates the membrane ester lipids, which are subsequently transferred to the blood plasma. It might well be that the intracellular enzyme reacted with free PUFAs and that the free oxygenated PUFAs are subsequently incorporated into the membrane ester lipids, which are then transferred to the blood plasma lipids. Both mechanistic scenarios are plausible, but it is difficult to differentiate between the two pathways.

**Fig. 8:**
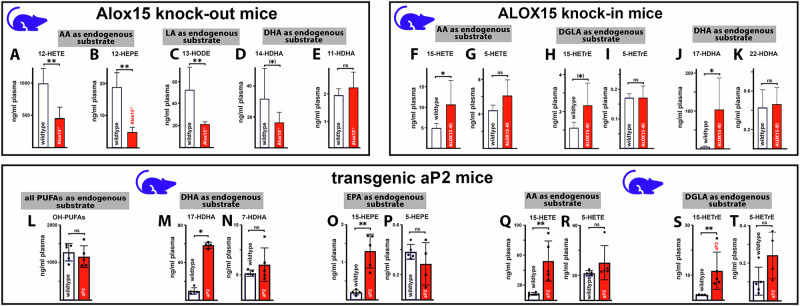
Presence of ALOX15-derived oxidized ester lipids in the blood plasma of genetically modified mice. **A–E** Presence of specific Alox15 products (12-HETE, 12-HEPE, 13-HODE, 14-HDHA) in the blood plasma lipids of wildtype mice (white bars) but their absence in Alox15^−/−^ animals (red bars). For 11-HDHA (no Alox15 metabolite) no difference was observed between the two genotypes. **F-K** Presence of specific ALOX15 products (15-HETE, 15-HETrE, 17-HDHA) in the blood plasma lipids of humanized Alox15 knock-in mice (red bars) but their absence in wildtype control animals (white bars). For the control metabolites (5-HETE, 5-HETrE, 22-HDHA) no significant differences between the two genotypes were observed. **L-T** Presence of specific Alox15 products (17-HDHA, 15-HEPE, 15-HETE, 15-HETrE) in the plasma lipids of wildtype mice (white bars) and of aP2 animals (red bars). For the control metabolites 7-HDHA, 5-HEPE, 5-HETE and 5-HETrE (no Alox15 metabolite) no differences were observed between the two genotypes. Five different individuals of each genotype were sacrificed (*n* = 5) and each sample was analyzed once.

First, we analyzed the plasma lipids of *Alox15*^−*/*−^ mice^57^ and expected that the levels of 12-HETE, 12-HEPE and 13-HODE, which constitute the major oxygenation products of mouse Alox15 catalyzed oxygenation of free AA, EPA and LA, are significantly lower in these animals than in wildtype controls. As indicated in Fig. 8A–C, corresponding data were actually obtained. For 14-HDHA (Fig. 8D), which is the major product of mouse Alox15-catalyzed DHA oxygenation, we observed a similar difference but for this metabolite only borderline significance was calculated (*p* = 0.058). In contrast, for 11-HDHA, which is not an Alox15 product, no difference was detected between the two genotypes (Fig. 8E).

Similar analyses were performed on the plasma lipids prepared from humanized Alox15-knock-in (Alox15-KI) mice. These animals express the AA 15-lipoxygenating Leu353Phe mutant of mouse Alox15 instead of the AA 12-lipoxygenating wildtype enzyme^58^. Here we expected that 15-HETE, 15-HETrE and 17-HDHA levels are significantly elevated in the Alox15-KI mice when compared with wildtype control animals. In contrast, for metabolites that are not related to the Alox15 pathway, such as 5-HETE, 5-HETrE, 22-HDHA, we should not detect significant differences between the two genotypes. This prediction was confirmed experimentally for 15-HETE, 15-HETrE and 17-HDHA (Fig. 8F, H, J). In contrast, for the control metabolites 5-HETE, 5-HETrE, 22-HDHA (Fig. 8G, I, K) no significant differences between Alox15-KI mice and wildtype control animals were observed.

Finally, we performed corresponding analyses with the plasma lipids of ALOX15-aP2 mice, which carry the human *ALOX15* gene in addition to the endogenous mouse *Alox15* gene. For these animals we expected elevated levels of 17-HDHA, 15-HEPE, 15-HETE and 15-HETrE in the plasma lipids. In contrast, there should be no significant differences for ALOX15-indipendent metabolites (7-HDHA, 5-HEPE, 5-HETE and 5-HETrE). As indicated in Fig. 8L no significant difference between the two genotypes was observed when the degree of oxygenation of all plasma lipids was compared and thus, transgenic expression of human ALOX15 did not lead to major oxidative stress that is mirrored on the level of the plasma lipids. However, as predicted significantly higher amounts of 17-HDHA, 15-HEPE, 15-HETE and 15-HETrE were analyzed in aP2 mice (Fig. 8M, O, Q, S). For the corresponding control metabolites (7-HDHA, 5-HEPE, 5-HETE and 5-HETrE) no differences between the two genotypes were observed (Fig. 8N, O, R, T).

## Discussion

The canonical scenario of the AA cascade suggests that ALOX-isoforms oxygenate free PUFAs, which have previously been liberated from complex membrane ester lipids^59,60^. *Via* this metabolic route, bioactive signal transducers are synthesized, which play a role as pro- and anti-inflammatory mediators^61^ but also regulate cancer cell proliferation and metastasis^62^. However, there are scattered reports in the literature suggesting that some ALOX isoforms are capable of oxidizing PUFA-containing phospholipids^18–20,23,24,63^ and these reactions have been implicated in cell differentiation^64^ and erythropoiesis^25^ but also in the pathogenesis of cardio-vascular^28^ and neurological diseases^65^. Unfortunately, there was no systematic study in the literature in which the catalytic activity of different ALOX-isoforms with PUFA-containing phospholipids was compared. To fill this gap, we explored the interaction of selected recombinant mammalian ALOX isoforms with different types of complex substrates and found that human ALOX15, mouse Alox15 and human ALOX15B are capable of oxygenating liposomes, mitochondrial and ER membranes as well as LDL and HDL. In contrast, the capability of mouse Alox15b and human ALOX12 was limited. Interestingly, erythrocyte membranes were not a substrate for any ALOX isoforms. For human ALOX15, mouse Alox15 and human ALOX15B the reaction specificity with PUFA-containing phospholipids was similar to that of free PUFA oxygenation but for mouse Alox15b a variable reaction specificity was observed.

The dominant formation of 13*S*-HODE-containing ester lipids by most mammalian ALOX isoforms could be predicted because of two reasons: i) LA is the dominant PUFA in most PUFA-containing ester lipids. ii) 13S-HODE is the dominant product of free LA oxygenation (Fig. 1). However, the dominant formation of 13*S*-HODE by mouse Alox15b with liposomes (Fig. K+L) was surprising since this enzyme oxygenates free LA dominantly to 9*S*-HODE (Fig. 1T). On the other hand, this finding was consistent with previously published data indicating that human/mouse ALOX15B orthologs convert AA-containing phospholipids mainly to the 15-HETE derivatives when the substrates were provided in the form of nanodiscs^36^. From this data, the authors concluded that human and mouse ALOX15B orthologs exhibits a similar reaction specificity when reacting with complex lipid-protein assemblies. This interpretation was of biological interest for redefining the role of the two enzyme orthologs in atherogenesis^36^. In our study, we confirmed this conclusion for liposomes, which as nanodiscs, constitute artificial substrate preparations (Fig. 3K, L). However, when we analyzed the reaction products formed by mouse Alox15b with naturally occurring mitochondrial (Fig. 4Q, R) and ER membranes (Fig. 5Q, R) we found that 9*S*-HODE was the major oxygenation product. From this data the following conclusions on the reaction specificity of mouse Alox15b with complex substrates can be drawn: i) Free LA and AA are converted by this enzyme to 9*S*-HODE and 8*S*-HETE, respectively. ii) When these PUFAs are provided in form of phospholipid liposomes (Fig. 3K, L) and/or phospholipid-containing nanodiscs^36^ esterified 13*S*-HODE and 15*S*-HETE are the major oxygenation products. iii) When esterified LA and AA are offered as substrates in the form of native biomembranes (Fig. 4Q, R, Fig. 5Q, R) 9*S*-HODE was dominant and thus, the product pattern resembles that of free LA oxygenation. The mechanistic basis for the variable reaction specificity of mouse Alox15b has not been explored in detail but a variable orientation of the substrates at the active site of the enzyme might contribute^36^.

This study was aimed at  characterizing the capabilities of selected mouse and human ALOX isoforms to oxygenate PUFA-containing phospholipids incorporated in liposomes, biomembranes and lipoproteins. We did not explore the phospholipid oxygenase activity of all functional human ALOX isoforms, but we selected the three most relevant human enzymes. The human genome involves 6 functional ALOX genes, but ALOX5 is not capable of oxidizing complex substrates^66,67^. ALOX12B and ALOXE3 orthologs are mainly expressed in the skin and they have previously been suggested to oxygenate epidermal ceramides contributing to the formation of the epidermal water barrier^64^. Unfortunately, recombinant expression of these two enzymes is problematic since the expression levels are rather low^68^. Moreover, the AA oxygenase activities of these two enzymes strongly depend on the reaction conditions^69–71^ and thus, our normalization strategy would not work with them. Mouse Alox12 exhibits a similar reaction specificity with free AA as its human ortholog, and thus, the results obtained here for human ALOX12 can be transferred to mouse Alox12.

In the present study, we routinely employed crude recombinant enzyme preparations. Human ALOX15, human ALOX15B and human ALOX12 could easily be purified from the cellular lysate supernatants, and selected experiments were performed with purified recombinant human ALOX15 (Fig. S6, S12, S14). However, for mouse Alox15, we experienced a dramatic loss in the catalytic activity during the purification procedure, which might be related to the observation that the iron content of the final enzyme preparation was rather low (20%). Moreover, mouse Alox15b did not firmly adhere to the affinity matrix and thus, no purification of this enzyme was possible. Because of these problems, we decided to employ crude enzyme preparations for our activity assays, but this strategy required additional control incubations (E. coli controls, Sf9 cell controls). In these control incubations, we added the cellular lysate supernatants of E. coli cells to the in vitro activity assays, which had been transformed with a non-recombinant expression plasmid. For human ALOX15 that was expressed in Sf9 insect cells, we used a cellular lysate supernatant of Sf9 cells, which were infected with a non-recombinant baculovirus.

For our liposome oxygenase activity assays, we prepared liposomes that mainly involve LA-containing phospholipids as ALOX substrates (Fig. 2). We selected these lipids since LA is the most abundant PUFA in most mammalian cells and tissues. However, in additional experiments, we also quantified the oxygenation efficiency of liposome preparation carrying other PUFA-containing phospholipids. Here we observed that not only the fatty acid composition (LA vs. AA vs. DHA) but also the phospholipid composition (PC vs. PE vs. PS) of the liposomes impacted the liposome oxygenase activity of native rabbit ALOX15. Whether this may also be the case for the other ALOX-isoforms will be tested in a follow-up study.

## Materials and methods

### Chemicals and devices

The chemicals and devices used in this study, including their sources, are summarized in Tables S3+4 of the Methodological Supplement.

### Software packages

Experimental raw data were evaluated using the Microsoft Excel software package (version 16/78). For preparation of the images the GraphPad Prism program (version 8.3.1. for macOS) and the Adobe Photoshop program (version 21.1.1, for macOS) were employed. For quantification of the band intensities in immunoblotting we used the ImageJ software package.

### Preparations

#### Preparation of small and large liposomes

Soybean phospholipids (800 µg, consisting of 38% phosphatidylcholine, 30% phosphatidylethanolamine, 18% phosphatidylinositol, 7% phosphatidic acid, 7% lysophosphatidyl choline, 0.1% butylated hydroxytoluene) and 200 µg bovine heart cardiolipin were dissolved in 500 µL of chloroform in a 25 mL cylindrical glass flask that was previously filled with argon gas. The flask was conneteced to a Heidolph VV200 vacuum rotatory evaporator that was rotated with a rate of 60 min^−1^ at  45 °C, and 0 mbar. The flask was removed after the chloroform had completely evaporated. Then, 500 µL of Dulbecco’s phosphate-buffered saline (DPBS) was added, the mixture was vortexed and then sonicated in an ultrasonic bath for 1 min. Subsequently, uniformly sized 100 nm liposomes were extruded. For this purpose, an Avanti Mini-Extruder was assembled with two 10 mm filter supports on each side of a polycarbonate membrane (0.1 µm pore size, for small liposomes). Before assembly, the filter membranes and support filters were wetted in DPBS. Two syringes were washed with DPBS, and one of them was filled with the liposome suspension. The syringes were connected to the extruder, and the liposome mixture was passed through the filtering device 30 times from one syringe to the other. The extruder temperature was maintained at 65 °C throughout the entire preparation procedure. Finally, the liposome suspension was removed from the extruder and transferred to a 1 ml Eppendorf tube filled with argon gas. The liposome suspension (2 mg lipids per ml suspension) was stored overnight at 4 °C and was employed as ALOX substrate to quantify the liposome oxygenase activity of different ALOX isoforms. For the preparation of large liposomes, a similar preparation procedure was employed, but here the initial liposome suspension was filtered through a polycarbonate membrane with a 1 µm pore size. Liposomes were freshly prepared for each experiment and were never stored longer than 24 h.

#### Preparation of beef heart mitochondrial membranes (electron transfer particles, ETP)

Electron transfer particles (ETP) constitute functional inside-out vesicles of beef heart mitochondrial membranes. They can be prepared from beef heart mitochondria as described in ref. ^72^. These membranes carry a functional respiratory chain as indicated by oxygraphic measurement of their NADH_2_-oxidase and succinate oxidase activities. To prepare these membranes, we obtained fresh beef heart from the local slaughterhouse and homogenized the tissue. From the tissue homogenates, the mitochondria were isolated by differential centrifugation. After sonication of the beef heart mitochondria, the membrane fragments were spun down, resuspended in DPBS and dialyzed overnight against DPBS. Finally, DPBS was added to the membrane suspension to reach a final membrane protein concentration of 20 mg membrane protein per mL, and aliquots of this suspension were shock-frozen in liquid nitrogen and stored at −80 °C.

#### Preparation of rat liver rough endoplasmic membranes (rER)

Rough endoplasmic membranes were prepared from rat liver according to the procedure described in ref. ^73^. In brief, the liver was prepared from sacrificed rats and the tissue was homogenized at 4°C in three volumes of an aqueous solution containing 0.25 M sucrose, 50 mM Tris-HCI, pH 7.4, 25 mM KCI, 5 mM MgCl_2_ and 2 mM dithiothreitol (DTT). Cell debris and mitochondria were removed by 10 min centrifugation at 10,000 x g and the post-mitochondrial supernatant was layered over a discontinuous sucrose gradient containing 1.25 mL of 1.3 M sucrose and 0.5 mL of 2.25 M sucrose both in the presence of 20 mM HEPES-KOH (pH 7.5), 1 mM MgCI_2_, and 2 mM DTT. The sucrose gradients were filled in 9-mL polycarbonate tubes and were centrifuged in an angle rotor for 1 h at 100,000 x g. Rough ER membranes, which were sedimented at the 1.3 M sucrose - 2.25 M sucrose interface, were pooled, dialyzed overnight against DPBS and adjusted to a final membrane protein concentration of 20 mg/ml. The membrane suspension was aliquoted, shock-frozen in liquid nitrogen and stored at −80 °C.

#### Preparation of human erythrocyte ghosts

Hemoglobin-free erythrocyte ghosts were prepared from human erythrocytes following the method of Dodge and Mitchel^74^. In brief, 200 ml of blood was drawn from a human volunteer and coagulation was prevented by the addition of 5 mM EDTA. Cells were spun down, the blood plasma and the buffy coat were removed by aspiration. The remaining red blood cells were washed three times with DPBS. Finally, the red cell pellet was resuspended for osmotic hemolysis at a dilution of 1:15 (v/v) in 20 mM phosphate buffer, pH 7.4. After a 1 h hemolysis period at 4 °C the erythrocyte ghosts were sedimented at 20,000 x g for 40 min, washed three times in DPBS and were finally dialyzed overnight against DPBS. The final membrane preparation was diluted with DPBS to reach a membrane-protein concentration of 20 mg/ml. Aliquots of the membrane suspension were shock-frozen in liquid nitrogen and stored at −80 °C until further use as ALOX substrates.

#### Preparation of human lipoproteins

Lipoproteins are spherical lipid-protein assemblies, which function as lipid transporters in the human blood. According to their density, four major lipoprotein classes exist in the human blood: i) chylomicrons (CM), ii) very low-density lipoprotein (VLDL), iii) low-density lipoprotein (LDL), iv) high-density lipoprotein (HDL). Human LDL and HDL have previously been reported to function as substrates for rabbit ALOX15^31^. To test whether other ALOX isoforms may also exhibit lipoprotein oxygenase activity, we prepared human LDL and HDL and used these preparations as ALOX substrates. In initial experiments, we employed commercial LDL and HDL preparations (Sigma-Aldrich, Saint Louis, USA), but these lipoprotein preparations showed OH-PUFA / PUFA ratios > 1%. These data indicated that the lipoprotein lipids of the commercial preparations were already highly oxidized and thus, these lipoprotein preparations could not be used as substrates for our ALOX preparations.

Thus, we decided to prepare fresh lipoproteins. For this purpose, we employed the sequential density gradient floating ultracentrifugation method that is described in detail in ref. 75 and here we provide a brief overview of the preparation steps. For our preparations, we obtained 200 mL of freshly prepared human plasma from the local blood bank and added solid potassium bromide (KBr) to reach a final density of 1.21 g/mL. This sample was centrifuged for 4 h at 100,000x g in 90 mL Quick-seal tubes (Beckman, Palo Alto, USA) in a Beckman LE80K ultracentrifuge. The upper yellow colored layer, which involved the floated lipoproteins, was recovered, and the different lipoprotein classes were subsequently separated in a second centrifugation step. For this purpose, a KBr step gradient was prepared in 10 mL centrifugation tubes of an SW41 swingout rotor. To prepare the step gradient, we first filled 1 mL of water into a 10 mL centrifugation tube and then the water was underlayered with 3.5 mL of a KBr solution exhibiting a density of 1.019 g/mL (10 mM Tris-HCL buffer containing 0.9% NaCl). Next, the 1.019 g/mL solution was underlayered with 3.5 mL of a denser KBr solution (1.063 g/mL, in 10 mM Tris-HCL buffer containing 0.9% NaCl) and finally 3 mL of the mixed lipoprotein solution of the first centrifugation step (density of 1.21 g/mL) were underlayered. These KBr step gradients were centrifuged for 20 h at 100,000 x g in a Beckman SW40Ti swing-out rotor. The orange-colored LDL fraction and the white-colored HDL fraction were recovered. These fractions were dialyzed overnight against 10 mM Tris-HCl buffer (pH 8.6) containing 150 mM NaCl and 1.5 mM EDTA, and the total protein concentrations were determined using the Bradford assay. HPLC analysis of the OH-PUFA / PUFA ratio of the lipoprotein preparations indicated an oxidation degree of the lipoprotein lipids of 0.04%. 0.5 mL aliquots of the dialyzed lipoprotein preparations were shock-frozen in liquid nitrogen and stored at −80 °C.

### Other methods

#### Cloning of mammalian ALOX isoforms

To test the capacity of selected human and mouse ALOX isoforms to oxygenate complex substrates, we first extracted cDNAs of the selected enzymes from genomic sequences retrieved from databases and constructed in silico an expression plasmid suitable for recombinant expression in E. coli. For this purpose, a *Sal*I restriction site was introduced immediately upstream of the ALOX cDNAs' start codons, and the original ATG was left unaltered. A *Hind*III recognition sequence was designed immediately downstream the stop codon and internal *Hind*III and *Sal*I sites were eliminated by silent nucleotide exchanges. Next, the cDNA sequences were optimized in silico for bacterial expression, and the constructs were chemically synthesized (Biocat GmbH, Heidelberg, Germany) into the multicloning site of the pUC57 cloning vector. For prokaryotic expression, the expression construct was excised from the synthesizing vector and subcloned into the multicloning site of the pET28b expression plasmid (Novagen/Merck, Darmstadt, Germany) between the SalI and HindIII restriction sites. Recombinant expression plasmids were tested for the presence of the ALOX inserts by *Sal*I + *Hind*III digestion, and the final expression constructs were completely sequenced (Eurofins Genomics Germany GmbH, Ebersberg, Germany).

#### Bacterial expression of ALOX isoforms

For this study we expressed the recombinant ALOX isoforms as described in ref. ^52^ with minor modifications. In brief, we transformed competent *E. coli* cells (pLysS Rosetta 2 DE) with the recombinant expression plasmids and the cells were grown overnight on agar plates containing antibiotics. Two separate bacterial clones were isolated and 1 mL bacterial liquid pre-cultures (LB medium with 50 μg/mL kanamycin and 35 µg/mL chloramphenicol) were grown overnight at 37 °C. Next, we added an appropriate volume of the pre-cultures to 50 mL sterile culture medium (Enpresso® B kit, Enpresso GmbH, Berlin, Germany) to adjust an OD_600_ of 0.1. After this, cells were cultured at 30 °C overnight so that the optical density at 600 nm has reached values > 5. Expression of the recombinant ALOX isoforms was induced with 1 mM (final concentration) of isopropyl-β-D-thiogalactopyranosid (IPTG). Booster tablets and reagent A (Ebpresso® B kit, Enpresso GmbH, Berlin, Germany) were added to speed up the expression process, and the liquid cultures were maintained at 22 °C for 18 h. Bacteria were spun down (4000 x g for 15 min), washed three times with 20 mL of DPBS and the cell pellet was reconstituted in a total volume of 10 mL DPBS containing 2 mM EDTA. Bacterial lysis was performed by sonication (Branson W-250P tip sonifyer, Heineman, Schwäbisch-Gmünd, Germany). Cell debris was removed (15 min, 15,000 x g, 4 °C), the cell-free lysis supernatants containing the recombinant ALOX isoforms were aliquoted (1 mL portions) and snap-frozen in liquid nitrogen.

#### Expression of human ALOX15 in Sf9 insect cells

Human ALOX15 was neither well expressed in E. coli nor in HEK293 cells, and thus, we decided to express the enzyme as N-terminal recombinant hexa-his-tag fusion protein in Sf9 insect cells. For this purpose, the ALOX15 coding region including the hexa-his tag peptide was excised from the recombinant bacterial expression plasmids and ligated into the pFastBac HT vector. The bacmids and the recombinant baculoviruses were generated according to the manufacturer’s instructions (Bac-to-Bac® Baculovirus Expression System, Invitrogen Life Technologies/ThermoFisher, Schwerte, Germany). Protein expression was initiated in *Sf9* cells (ThermoFisher, Schwerte, Germany) using the Insect XPRESS Medium (Biozym Scientific GmbH, Hessisch Oldendorf, Germany) supplemented with 4 mM glutamine and 0.5% FCS. The cells were infected with a DOI of 1 and subsequently incubated at 27 °C and 120–130 rpm on an agitation platform. After 72 h of incubation (30% dead cells), the cells were harvested by centrifugation, lysed by sonication (Branson W-250P tip sonifyer, Heineman, Schwäbisch-Gmünd, Germany) and the cell-free lysate supernatant was used as enzyme source.

#### Purification of recombinant human ALOX15

2 ml of the cellular lysis supernatant of human ALOX15 expression was mixed with 1.5 mL of Ni-NTA resin (Invitrogen, Toulouse, France) for 1 h at 4 °C. The resin with the bound his-tag fusion proteins was transferred to an open bed 10-mL plastic column. The column was washed two times with 2 mL of washing buffer I (100 mM Tris, pH 8.0, 200 mM NaCl, 10 mM imidazole) and two times with 2 mL of washing buffer II (100 mM Tris, pH 8.0, 200 mM NaCl, 25 mM imidazole). Finally, the adhering his-tag fusion proteins were eluted five times with 0.6 mL elution buffer (100 mM Tris-HCl, pH 8.0, 200 mM NaCl, 200 mM imidazole). Enzyme containing elution fractions were combined, the enzyme solution was desalted using an Econo-Pac 10DG column (Bio Rad, Munich, Germany) and recombinant ALOX15 was further purified by conventional anion exchange chromatography. For this purpose, an FPLC-system (GE Healthcare Bio-Sciences AB, Uppsala, Sweden) equipped with a Resource Q 6-mL column was used and adhering proteins were eluted by a linear NaCl gradient. 20 mM Tris (pH 6.8 or 8.0) was used as elution buffer A and 20 mM Tris/1 M NaCl (pH 6.8 or 8.0) as buffer B. The fractions containing active ALOX protein were pooled, desalted and homogeneity of the samples was checked by SDS-PAGE. The final enzyme preparation was stored at −80 °C until use.

#### SDS-PAGE and quantitative Western blotting

To quantify the level of enzyme expression, aliquots (2–20 µL) of the cellular lysis supernatants were analyzed by SDS-PAGE on a 7.5% polyacrylamide gel. Separated proteins were transferred to a 0.45 µm nitrocellulose membrane (Serva GmbH, Heidelberg, Germany) by a wet-blotting method. The membranes were blocked with blocking solution (Serva GmbH, Heidelberg, Germany), washed three times with DPBS containing 0.3% TWEEN 20 and were finally incubated with an anti-His-HRP antibody (ThemoFisher, Schwerte, Germany) for 1 h at room temperature. After several steps of washing, the membrane was stained using the SERVALight Polaris CL HRP WB Substrate Kit (SERVA GmbH, Heidelberg, Germany) for 5 min at room temperature. Chemiluminescence was quantified using the FUJIFILM Luminescent Image Analyzer LAS-1000plus (Fujifilm Europe GmbH, Düsseldorf, Germany). For quantification of the luminescence intensity, different amounts of purified *M. fulvus* ALOX15^76^ were applied to immunoblotting, which was also expressed as N-terminal hexa-his-tag fusion protein.

#### Measurement of the in vitro free fatty acid oxygenase activity

To quantify the free polyenoic fatty acid oxygenase activities of the different lipoxygenase preparations, aliquots of the cellular lysis supernatants (0.5–5 µL) were incubated in 0.5 mL of DPBS containing 100 µM of substrate fatty acids (arachidonic acid, linoleic acid) for 3 min at room temperature in 2 ml Eppendorf tubes^48^. The ALOX reaction was terminated by the addition of 1 mg solid sodium borohydride, which inactivates the enzyme (reduction to the catalytically silent ferrous form) and reduces the hydroperoxy fatty acids formed during the ALOX reaction to the more stable alcohols. After a 5 min incubation on ice, 35 µL of glacial acetic acid were added to acidify the sample. Then the cellular lysate proteins were precipitated by the addition of 0.5 mL of acetonitrile, and following a 15 min incubation period on ice, the protein precipitate was spun down. The supernatant was recovered, and aliquots were analyzed by RP-HPLC for the formation of ALOX products. For control purpose, three different types of control incubations were carried out: i) DPBS control: In these incubations, 5 µL of DPBS was added to the incubation mixture instead of the cellular lysate supernatants. In these incubations, we quantified the oxygenation products formed during the incubation period via autoxidation. ii) Non-recombinant cell lysate supernatant: In these incubations, aliquots of cellular lysate supernatants were added to the incubation mixture, which were generated by transforming E. coli cells with a non-recombinant expression plasmid (bacterial expression) or by infecting Sf9 cells with the non-recombinant baculovirus (Sf9 cell expression). iii) Denatured lysate supernatant: In these incubations, the aliquots of the cellular lysate supernatants were heated to 90 °C for 3 min before they were added to the incubation mixture. We found that in all of these control incubations, the formation of oxygenated PUFA derivatives was minimal.

#### Normalization of the different enzyme preparations

To quantify the relative liposome oxygenase activities of the different enzyme preparations, we normalized them for the same AA oxygenase activity. For this purpose, we first assayed the AA oxygenase activity of the cellular lysate supernatants by adding equal volumes (2 µL) of the cell-free lysate supernatants to the AA oxygenase assay. Next, we quantified by RP-HPLC the oxygenation products formed during a 3 min incubation period and this measure was used as a readout parameter for the AA oxygenase activity of the enzyme preparations. Finally, we calculated the lysate supernatant volumes which correspond to the same arachidonic acid oxygenase activity and these volumes were added to the in vitro liposome, in vitro biomembrane and in vitro lipoprotein oxygenase activity assays.

#### In vitro liposome oxygenase activity assays

To quantify the liposome oxygenase activities, we first normalized the different enzyme preparations with respect to their arachidonic acid oxygenase activity. For enzyme preparations exhibiting high arachidonic acid oxygenase activity, smaller volumes of cellular lysate supernatant were used to perform the liposome oxygenase activity assay. In contrast, for enzyme preparations with lower arachidonic acid oxygenase activity, larger volumes were employed. The liposome activity assays were carried out in 2 mL Eppendorf tubes, which were first filled with 0.5 mL of DPBS containing small or large liposomes at a concentration of 0.5 mg liposomal lipids per ml DPBS. The reaction was started by the addition of aliquots of the enzyme preparations exhibiting a similar arachidonic acid oxygenase activity. After a 15 min incubation period at room temperature, the ALOX reaction was terminated by the addition of 1 mg solid sodium borohydride, which inactivated the enzyme (reduction to the catalytically silent ferrous form) and simultaneously reduced the hydroperoxy fatty acids formed during the ALOX reaction to the more stable alcohols. After a 5 min incubation on ice, 35 µL of glacial acetic acid were added to acidify the sample. Then the cellular lysate proteins were precipitated by the addition of 0.5 mL of acetonitrile, and following a 15 min incubation period on ice, the protein precipitate was spun down. The supernatant was recovered, the total lipids were extracted^77^ and the ester lipids were hydrolyzed under alkaline conditions. After the hydrolysis procedure, the samples were acidified by the addition of 75 µL of glacial acetic acid, precipitate was removed by centrifugation (20,000 x g for 15 min at 4 °C) and aliquots of the acidified sample were analyzed by RP-HPLC to quantify the amounts of the hydroxy PUFA (following the chromatogram at 235 nm) and of the non-oxidized substrate PUFAs (following the absorbance at 210 nm). For control purpose, four different types of incubations were carried out: i) DPBS control: In these incubations 5 µL of DPBS were added to the incubation mixture instead of the cellular lysate supernatants. ii) Non-recombinant cell lysate supernatant: In these incubations, aliquots of cellular lysate supernatants were added to the incubation mixture, which were generated by transforming the E. coli cells with a non-recombinant expression plasmid (bacterial expression) or by infecting Sf9 cells with the non-recombinant baculovirus (Sf9 cell expression). iii) Denatured lysate supernatant: In these incubations, the aliquots of the cellular lysate supernatants were heated to 90°C for 3 min before they were added to the incubation mixture. iv) Non-hydrolyzed lipid extracts: To prove that the hydroxy PUFAs detected in the hydrolyzed lipid extracts originated from the cellular ester lipids but not from contaminating free fatty acids, we also analyzed the non-hydrolyzed lipid extracts. In all of these control incubations, the amounts of oxygenated PUFAs were minimal.

#### In vitro membrane oxygenase activity assays

To quantify the membrane oxygenase activities, we also normalized the different enzyme preparations with respect to their arachidonic acid oxygenase activity, as described for the measurements of the liposome oxygenase activities. Here, we followed the basic experimental protocol described in ref. ^20^ with minor modifications. The membrane oxygenase activity assays were carried out in 2 mL Eppendorf tubes, which were first filled with 0.5 ml of DPBS containing either mitochondrial or endoplasmic membranes as ALOX substrate at a concentration of 1 mg membrane protein per ml DPBS. The reaction was started by the addition of aliquots of our enzyme preparations exhibiting a similar arachidonic acid oxygenase activity. After a 15 min incubation period at room temperature, the ALOX reaction was terminated by the addition of 1 mg solid sodium borohydride, which inactivated the enzyme (reduction to the catalytically silent ferrous form) and simultaneously reduced the hydroperoxy fatty acids formed during the ALOX reaction to the more stable alcohols. Further sample work-up and RP-HPLC analysis of the oxygenation products were performed as described above for the in vitro liposome oxygenase activity assays.

#### In vitro lipoprotein oxygenase activity assays

To quantify the lipoprotein oxygenase activities of the enzyme preparations, we also normalized the different enzyme preparations to their arachidonic acid oxygenase activity (see Liposome oxygenase activity). Human low-density lipoprotein (LDL) and high-density lipoprotein (HDL) were prepared as described above^75^ and were immediately used as ALOX substrates. Repeated freezing and thawing were avoided since we observed autoxidation of the lipoprotein lipids. The lipoprotein oxygenase activity assays were carried out in 2 mL Eppendorf tubes, which were first filled with 0.5 ml of DPBS containing either freshly prepared human LDL or human HDL as ALOX substrate at a concentration of 1 mg apolipoprotein per ml DPBS. The reaction was initiated by the addition of aliquots of enzyme preparations exhibiting similar arachidonic acid oxygenase activity. Further sample work-up and RP-HPLC analysis of the oxygenation products were performed as described above for the in vitro liposome oxygenase activity assays.

#### In vitro hemolysis assays

ALOX-catalyzed oxygenation of the membrane lipids of intact erythrocytes induces hemolysis^52^. In other words, the degree of hemolysis can be quantified as suitable measure for the capability of a given ALOX isoform to oxygenate the membrane lipids of erythrocyte membranes. For our study, we incubated intact human erythrocytes with the recombinant ALOX isoforms and quantified the degree of hemolysis after short-term (15 min) and long-term (4 h) incubations. For this purpose, we followed the experimental protocol described in ref. ^52^ using the recombinant ALOX15 of P. aeruginosa as enzyme. In brief, 0.1 ml of packed human erythrocytes were incubated in 1 ml DPBS at room temperature in the presence or absence of different mammalian ALOX isoforms for different time periods. After the incubation periods, the cells were spun down, the supernatant was recovered and the hemoglobin content of the supernatant was determined measuring the absorbance at 410 nm (Soret band). For control purpose, no-enzyme control incubations were performed. 100% hemolysis was achieved when the erythrocytes were incubated for 45 min in 1 mL of ice-cold water.

#### Total lipid extraction

To extract the total lipids from the biological samples (liposomes, mitochondrial membranes, endoplasmic membranes, lipoproteins), we followed the classical method of Bligh and Dyer^77^. After borohydride reduction and acidification, 0.5 mL of DPBS were added to the activity assays, the samples were transferred to 20 mL glass tubes and were kept on ice for about 10 min. Then 2.5 mL of ice-cold methanol and 1.25 mL of chloroform were added and the samples were vortexed for 1 min (no phase separation). After 15 min on ice 1.25 mL of DPBS and 1.25 mL of chloroform were added and the samples were vortexed again for 1 min. For proper phase separation, the glass tubes were centrifuged for 10 min at 5000 x g and the lower phase containing the extracted lipids was recovered. The solvents were evaporated and the remaining lipids were reconstituted in 500 µL of methanol.

#### Alkaline hydrolysis of ester lipids

75 µL of anaerobic 40% KOH were added to the solution of the extracted lipids, the glass vials were filled with argon gas and were closed with glass stoppers and parafilm. Then the samples were incubated for 20 min at 60°C, which saponifies the ester lipids. After cooling down on ice, the samples were acidified and the drop of pH was confirmed by a pH indicator paper. Precipitate was spun down and aliquots of the ester lipid hydrolysates were analyzed by RP-HPLC to quantify the amounts of OH-PUFAs (following the chromatograms at 235 nm) and of the non-oxidized PUFAs (following the chromatogram at 210 nm).

#### RP-HPLC analysis of the polyenoic fatty acids and their oxygenated derivatives

To quantify the amounts of polyenoic fatty acids and their hydroxylated derivatives in the hydrolyzed lipid extracts, we routinely employed RP-HPLC with UV detection. For this purpose, aliquots of the hydrolyzed lipid extracts were injected into a Shimadzu HPLC system equipped with a SIL-20AC auto-injector. The analytes were separated isocratically on a EC 250/4 Nucleodur 100-5 C18 column (Macherey-Nagel, Düren, Germany) connected with an EC 4/3 Nucleodur 100-5 C18 ec pre-column (Macherey-Nagel, Düren, Germany) at room temperature using a solvent flow rate of 1 mL/min. A mobile phase consisting of acetonitrile : water : acetic acid (70 :30 : 0.1) was used. The UV-absorbancies at 210 nm (polyenoic fatty acids) and 235 nm (oxygenated polyenoic fatty acids) were simultaneously recorded and complete uv-spectra of the analytes were stored every 0.3 s. For quantitative purpose, the intensity scale of the chromatographic device was calibrated by injecting known amounts of the analyzed polyenoic fatty acids and of 15-HETE. The latter metabolite represents all conjugated dienes (12-HETE, 8-HETE, 13-HODE, 9-HODE, 15-HETrE, 13-HOTrE) since the molar extinction coefficient was identical for these compounds. For each metabolite 5–7 point calibrations curves were established.

#### Combined normal phase / chiral phase HPLC

Monohydroxylated PUFAs are optically active compounds, and they occur in two enantiomers, R- vs. S-isoforms. Racemic mixtures of R- and S-isomers are usually formed by autoxidation, but ALOX products are overwhelmingly chiral. Thus, quantification of the S/R-ratio of the oxygenated polyenoic fatty acids allows mechanistic conclusions on the metabolic origin of these compounds. Unfortunately, hydroxy fatty acid enantiomers can neither be separated by conventional RP- nor SP-HPLC. For isocratic enantiomer separation, HPLC columns with a chiral stationary phase are required. To separate hydroxy polyenoic fatty acid enantiomers, we employed a Diacel (Osaka, Japan) Chiralpak AD-H column (250 × 4.6 mm, 5 µm particle size). A mobile phase consisting of n-hexane : methonol : ethanol and acetic acid (96 : 3 : 1: 0.1, by vol) was used. For preparation of the mobile phase, we first mixed 30 ml of methanol with 10 ml of ethanol and 1 mL of acetic acid and added this solvent mixture to 960 ml of n-hexane. The solvent mixture was sonicated in a Sonorex Super RK512H ultrasonic bath (Bandelin GmbH, Berlin, Germany) and used the same day. It should be stressed that the solvent should be prepared each day freshly, since overnight storage of the solvent significantly elevated the retention times of the analytes. The solvent flow rate was set to 1.5 ml /min. The UV-absorbance at 235 nm (conjugated dienes) was recorded. The chemical identity of the analytes was determined by comparison of the retention times with authentic standards and by UV-spectroscopy.

#### Blood plasma preparation

For quantification of the oxygenated fatty acids in the blood plasma ester lipids EDTA blood was drawn from euthanized mice by cardiac puncture. For this purpose, the mice were anesthetized by inhalation of isoflurane and the isoflurane concentration in the breathing air was gradually increased over a time period of 5 min. After the mice had stopped breathing, they were taken out of the inhalation chamber and were sacrificed by cervical dislocation. Then, the heart was punctured, EDTA blood was drawn, and the blood cells were pelleted by centrifugation (500 x g for 15 min). The blood cells were discarded, and aliquots of the blood plasma were used for oxylipidome analyses. We have complied with all relevant ethical regulations for animal use and the approval number of the local animal care committee of Charité is T-CH 0003/22.

#### LC-MS/MS analysis of the blood plasma oxylipidomes

10 µL of blood plasma was mixed with 450 µl of water and 10 µl of a mixture of external standards (LTB4-d4, 20-HETE-d6, 15-HETE-d8, 13-HODE-d4, 14,15-DHET-d11, 9,10-DiHOME-d4, 12,13-EpOME-d4, 8,9-EET-d11, PGE2-d4; 10 ng/ml each). 5 µl of a butylhydroxytoluene (BHT) solution was added to prevent PUFA autooxidation during sample workup and storage. Plasma proteins were precipitated by the addition of 100 µl of a 1:4 mixture (by vol.) of glycerol/water and 500 µl acetonitrile. The pH was adjusted to 6.0 by the addition of 2 ml phosphate buffer (0.15 M), the precipitated proteins were removed by centrifugation and the clear supernatant was used for solid-phase lipid extraction on a 200 mg Agilent Bond-Elute-Certify II cartridge (Agilent Technologies, Santa Clara, USA). Before sample application, the cartridge was conditioned with 3 ml methanol and 3 ml phosphate buffer (0.15 M, pH 6.0). After the sample was applied, the column was washed with 3 ml of a 1:1 mixture (by vol.) of methanol : water and the oxygenated fatty acids were eluted with a 74 : 25 : 1 mixture (by vol.) of ethyl acetate : n-hexane : acetic acid. The solvents were evaporated in a stream of nitrogen, the remaining lipids were reconstituted in 100 µl of a 6:4 mixture (by vol.) of methanol : water and used for LC-MS/MS analysis. The chemical identity of the different analytes was concluded from co-chromatography with authentic standards, and for each of the quantified metabolites a calibration curve was set up.

LC-MS/MS was carried out on an Agilent 1290/II LC-MS system consisting of a binary pump system, an autosampler and a column oven (Agilent Technologies, Waldbronn, Germany). As stationary phase, we employed an Agilent Zorbax Eclipse C_18_ UPLC column (150 ×2.1 mm, 1.8 µm particle size). The column temperature was set at 30 °C. As mobile phase, we used a solvent gradient that was mixed of two stock solutions. Stock A: Water containing 0.05% acetic acid. Stock B: 1:1 mixture (by vol.) of methanol : acetonitrile. The HPLC system was connected with a triple quadrupole MS system (Agilent 6495 System, Agilent Technologies, Santa Clara, USA). Negative electrospray ionization was carried out. The mass spectrometer was run in dynamic MRM mode, and each metabolite was detected simultaneously by two independent mass transitions characteristic of the different analytes. Experimental raw data were evaluated with the Agilent Mass-Hunter software package, version B10.0. For all metabolites analyzed in this study, individual calibration curves were set up and the lower detection limits were also determined. A more detailed description of the analytical procedure is given in ref. ^78^.

#### Molecular docking and molecular dynamics (MD) simulation studies

To explore the binding mode of phospholipid substrates within the active sites of ALOX-isoforms molecular docking studies were carried out. For this purpose, the crystal structures of rALOX15 (PDB ID: 2P0M)^79^ and hALOX15B (PDB ID: 4NRE)^80^ were used. The 3D structure of the substrate molecule [1-stearoyl-2-arachidonoyl-sn-glycero-3-phosphocholine (SAPC)] was retrieved from the PubChem database (https://pubchem.ncbi.nlm.nih.gov). The geometry of SAPC was optimized using the GAUSSIAN 16 software package with the B3LYP functional and the 6–31 G basis set to obtain a stable conformation prior to docking. Before the docking procedure, all crystallographic water molecules were removed from the protein structures. A 10 Å spherical grid was generated around the centroid of the native ligand present in each crystal structure to define the docking site. Molecular docking was performed using the Genetic Optimization for Ligand Docking (GOLD) software^81^ with default parameters. To explore the ligand’s conformational flexibility within the binding pocket, 100 genetic algorithm (GA)-based docking runs were carried out. In both protein systems, a distance constraint of 3 Å was applied between the C13 atom of the sn-2 chain of SAPC and the non-heme iron atom to preserve a catalytically relevant orientation. The resulting docked poses were visualized and analyzed using BIOVIA Discovery Studio Visualizer. In the present study, we observed three distinct docking poses of the SAPC molecule inside the active site of two proteins (Figure S13). For further investigations, we selected pose 3 (Figure S13), in which the polar head group of the phospholipid molecule was oriented toward a solvent-exposed region of the enzyme, while the hydrophobic fatty acyl chains were localized inside the active site cavity.

To test the stability of the docking poses and to structurally optimize the enzyme–substrate complexes MD simulations were performed using Desmond software package (version 2025-1). For this purpose, the systems were solvated in an orthorhombic water box and neutralized with 0.15 M NaCl to mimic physiological conditions. Initial energy minimization was carried out for 250 ps to eliminate steric clashes and to optimize the system geometry. This procedure was followed by equilibration under an isothermal–isobaric (NPT) ensemble to ensure thermodynamic stability. Subsequently, a 100 ns MD simulation was conducted to examine the kinetic behavior of the enzyme-substrate complexes and to conclude the major interactions between the phospholipid substrate and the enzyme protein. This data is visualized in the supplemental movies M1 and M2.

#### Data repetitions and statistic evaluation

This study was designed to answer the following two questions: i) Which of the tested human and mouse ALOX isoforms are capable of oxygenating complex ester lipids, when these substrates are offered as complex structures such as liposomes, biomembranes or lipoproteins. ii) Which of the tested ALOX isoforms is best in catalyzing the oxygenation of complex substrates, and are there significant differences between the tested isoforms? To answer the first question, the difference between the series of measurements for the enzyme-dependent oxygenation rate and the corresponding control incubations was tested using the one-sided unpaired t-test. The null hypothesis was rejected for a *p* < 0.05. Lack of statistical significance is marked with ns in tables and figures. To answer the second question, the statistical significance of the differences between two series of measurements of enzyme-dependent oxygenation rates was calculated using the one-sided unpaired t-tests. The null hypothesis was rejected for a pB < 0.05. pB is the Bonferoni-corrected p-value required for multiple testing, pB = pxN(N-1)/2 (N = number of enzymes compared). To determine the enzyme ranking for a given substrate, a score was assigned to the variables to be compared (enzymes or substrates). This score is initially set to zero and increases or decreases by 1 if the measurement series of A and B differ from each other with pB < 0.05: s(A)=s(A) + 1, s(B)=s(B)-1 if A < > B with pB<0.05 and mean value (A) > mean value(B). The calculated total score is the basis for the ranking, whereby the variable with the highest score is assigned rank 1. Variables with the same score are given the same rank.

### Reporting summary

Further information on research design is available in the Nature Portfolio Reporting Summary linked to this article.

## Supplementary information

Supplementary Information

Description of Additional Supplementary Files

Supplementary Data 1

Supplementary Data 2

Supplementary Movie 1

Supplementary Movie 2

Reporting Summary

Transparent Peer Review file

## Data Availability

All data generated or analyzed during this study are included in this published article and/or in the online supplement. Original experimental raw data have been deposited on the servers of the two research institutions (Charité, Indian Institute of Petroleum and Energy) and can be obtained upon request from XC, HK. and PA. The original Western blot used for construction of Fig. 1A including the molecular weight markers is shown in the Supplementary information (Fig. S17). The original numeric experimental raw data used for construction of the bar diagrams are given as Excel files (Complex-substrates-data.xlsx, Modified-mice-data.xlsx) in the Supplementary Information.
