# Supplementary material for: Reactivity of mammalian lipoxygenases (ALOX isoforms) with phospholipids, biomembranes and lipoproteins
Source: Commun Biol. 2026 Jun 8;9:774. doi: 10.1038/s42003-026-10233-9 (PMC13246792; doi:10.1038/s42003-026-10233-9)
Supplement: Supplementary file 1 — Supplementary Information [file 42003_2026_10233_MOESM1_ESM.pdf]

Supplementary information to the paper

Reactivity of mammalian lipoxygenases (ALOX isoforms) with phospholipids, biomembranes and lipoproteins.

Xin Chen[1], Sarah Melissa Strätker[1], Sahanawaz Parvez[2], Ramunas Martin Vabulas, Astrid Bochert[1], Michael Rothe[3], Hermann-Georg Holzhütter[1], Polamarasetty Aparoy[2] and Hartmut Kuhn[1]*

[1]Charité - Universitätsmedizin Berlin, Corporate member of Freie Universität Berlin and Humboldt Universität zu Berlin, Department of Biochemistry, Charitéplatz 1, D-10117 Berlin, Germany.

[2]Molecular Modeling and Protein Engineering Lab, Biology Division, Department of Humanities and Sciences, Indian Institute of Petroleum and Energy, Visakhapatnam, Andhra Pradesh, 530003, India

[3]Lipidomix GmbH, Robert-Roessle-Str. 10, 13125 Berlin, Germany.

Running title: ALOX activity with complex substrate

Keywords: eicosanoids, liposomes, cell differentiation, ferroptosis, inflammation, atherosclerosis,

***Address correspondence to:** hartmut.kuehn@charite.de

1. Supplemental data

Figure S1. AA oxygenase activity of recombinant human ALOX15 expressed in Sf9 insect cells. Human ALOX15 was expressed as recombinant N-terminal his-tag fusion protein in Sf9 cells and the cellular lysate supernatant was used as enzyme source. In vitro activity assay and RP-HPLC analysis were carried out as described in the STAR Method section. Heat inactivation was induced by heating the lysate supernatant for 5 min at 90° C.

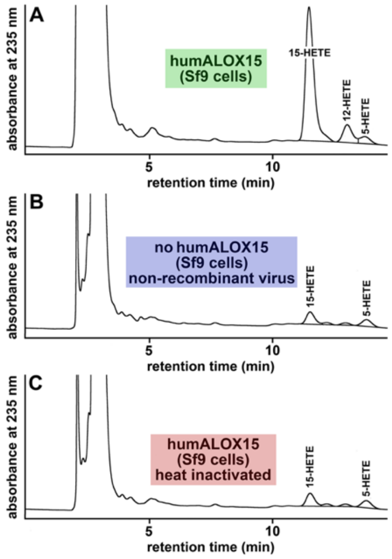

Figure S2. AA oxygenase activity of recombinant mouse Alox15 expressed in *E. coli*. Mouse Alox15 was expressed as recombinant N-terminal his-tag fusion protein in *E. coli* and the cellular lysate supernatant was used as enzyme source. In vitro activity assay and RP-HPLC analysis were carried out as described in the STAR Method section. Heat inactivation was induced by heating the lysate supernatant for 5 min at 90°C.

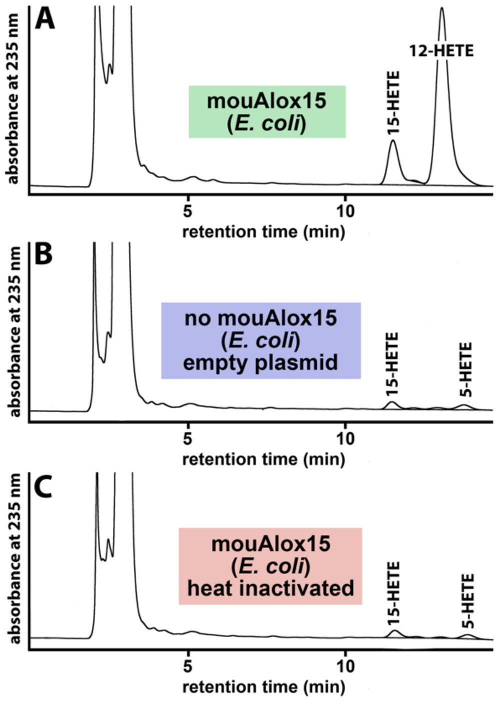

Figure S3. AA oxygenase activity of recombinant human ALOX15B expressed in *E. coli*. Human ALOX15 was expressed as recombinant N-terminal his-tag fusion protein in *E. coli* and the cellular lysate supernatant was used as enzyme source. In vitro activity assay and RP-HPLC analysis were carried out as described in the STAR Method section. Heat inactivation was induced by heating the lysate supernatant for 5 min at 90° C.

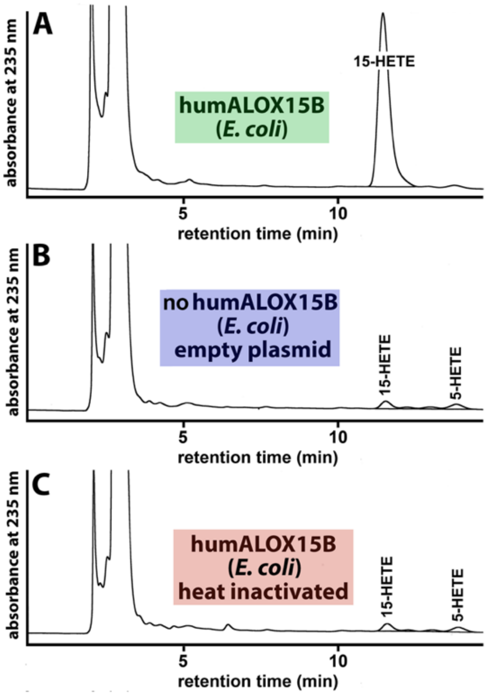

Figure S4. AA oxygenase activity of recombinant mouse Alox15b expressed in *E. coli*. Mouse Alox15b was expressed as recombinant N-terminal his-tag fusion protein in *E. coli* and the cellular lysate supernatant was used as enzyme source. In vitro activity assay and RP-HPLC analysis were carried out as described in the STAR Method section. Heat inactivation was induced by heating the lysate supernatant for 5 min at 90°C.

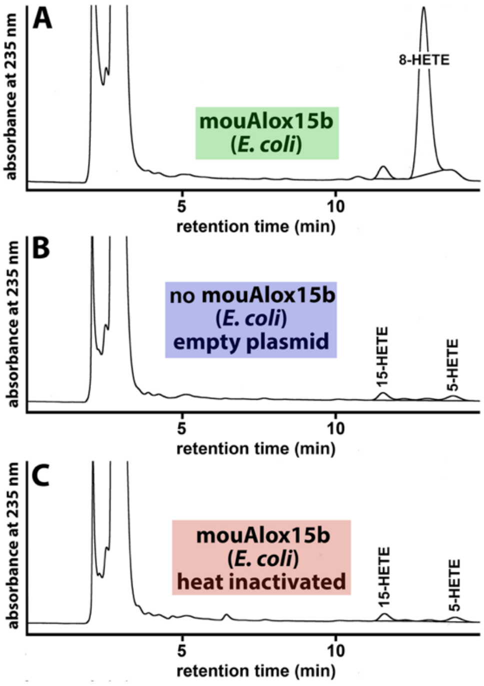

Figure S5. AA oxygenase activity of recombinant human ALOX12 expressed in *E. coli*. Human ALOX12 was expressed as recombinant N-terminal his-tag fusion protein in *E. coli* and the cellular lysate supernatant was used as enzyme source. In vitro activity assay and RP-HPLC analysis were carried out as described in the STAR Method section. Heat inactivation was induced by heating the lysate supernatant for 5 min at 90° C.

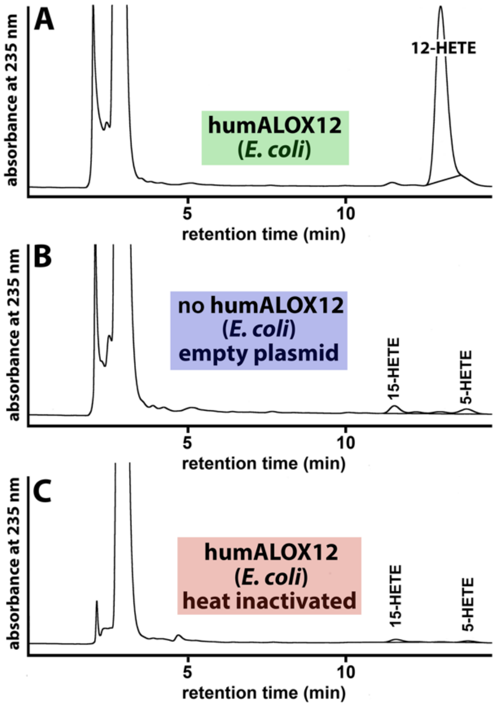

Figure S6. AA oxygenase activity of pure recombinant human ALOX15 expressed in Sf9 cells. Human ALOX15 was expressed as recombinant N-terminal his-tag fusion protein in Sf9 cells and purified to apparent electrophoretic homogeneity by affinity chromatography on Ni-agarose. Methodological details about enzyme preparation, in vitro activity assay and RP-HPLC analysis of the reaction products were carried out as described in the STAR Method section. Heat inactivation was induced by heating the lysate supernatant for 5 min at 90° C.

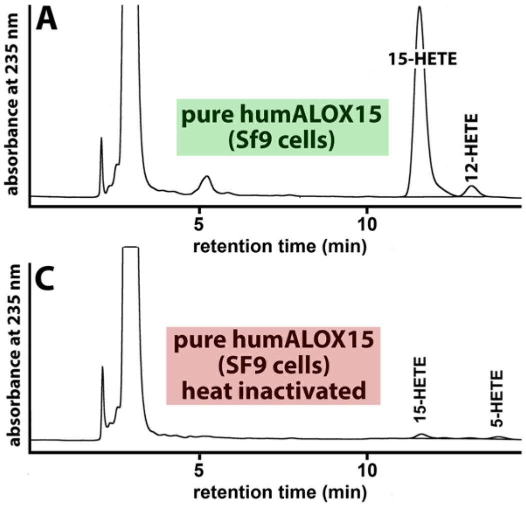

Figure S7. Liposome oxygenase activity of recombinant human ALOX15 expressed in Sf9 insect cells. Human ALOX15 was expressed as recombinant N-terminal his-tag fusion protein in Sf9 cells and the cellular lysate supernatant was used as enzyme source. Liposome oxygenase activity assays and RP-HPLC analysis of the reaction products were carried out as described in the STAR Method section. Heat inactivation was induced by heating the lysate supernatant for 5 min at 90° C. For the non-hydrolyzed samples an aliquot of the non-hydrolyzed lipid extract was directly injected to HPLC. The remaining sample was hydrolyzed. Since LA was present the dominant PUFA in the liposome preparations no HETE-isomers were analyzed.

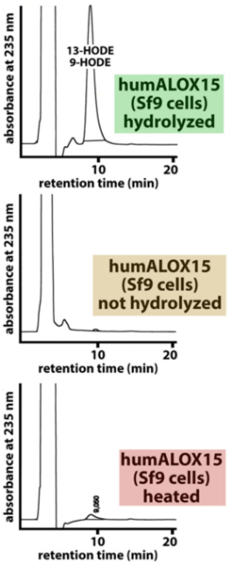

Figure S8. Liposome oxygenase activity of recombinant mouse Alox15 expressed in E. coli. Mouse Alox15 was expressed as recombinant N-terminal his-tag fusion protein in E. coli cells and the cellular lysate supernatant was used as enzyme source. Liposome oxygenase activity assays and RP-HPLC analysis of the reaction products were carried out as described in the STAR Method section. Heat inactivation was induced by heating the lysate supernatant for 5 min at 90° C. For the non-hydrolyzed samples an aliquot of the non-hydrolyzed lipid extract was directly injected to HPLC. The remaining sample was hydrolyzed. Since LA was the dominant PUFA in the liposome preparations no HETE-isomers were analyzed.

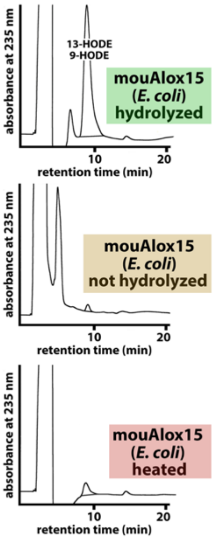

Figure S9. Liposome oxygenase activity of recombinant human ALOX15B expressed in E. coli. Human ALOX15B was expressed as recombinant N-terminal his-tag fusion protein in E. coli cells and the cellular lysate supernatant was used as enzyme source. Liposome oxygenase activity assays and RP-HPLC analysis of the reaction products were carried out as described in the STAR Method section. Heat inactivation was induced by heating the lysate supernatant for 5 min at 90° C. For the non-hydrolyzed samples an aliquot of the non-hydrolyzed lipid extract was directly injected to HPLC. The remaining sample was hydrolyzed. Since LA was the dominant PUFA in the liposome preparations no HETE-isomers were analyzed.

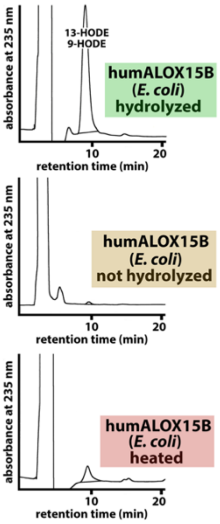

Figure S10. Liposome oxygenase activity of recombinant mouse Alox15b expressed in E. coli. Mouse Alox15b was expressed as recombinant N-terminal his-tag fusion protein in E. coli cells and the cellular lysate supernatant was used as enzyme source. Liposome oxygenase activity assays and RP-HPLC analysis of the reaction products were carried out as described in the STAR Method section. Heat inactivation was induced by heating the lysate supernatant for 5 min at 90° C. For the non-hydrolyzed samples an aliquot of the non-hydrolyzed lipid extract was directly injected to HPLC. The remaining sample was hydrolyzed. Since LA was the dominant PUFA in the liposome preparations no HETE-isomers were analyzed.

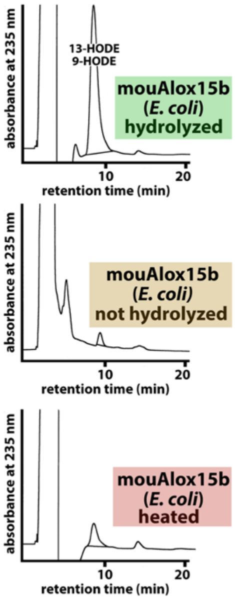

Figure S11. Liposome oxygenase activity of recombinant human ALOX12 expressed in E. coli. Human ALOX12 was expressed as recombinant N-terminal his-tag fusion protein in E. coli cells and the cellular lysate supernatant was used as enzyme source. Liposome oxygenase activity assays and RP-HPLC analysis of the reaction products were carried out as described in the STAR Method section. Heat inactivation was induced by heating the lysate supernatant for 5 min at 90° C. For the non-hydrolyzed samples an aliquot of the non-hydrolyzed lipid extract was directly injected to HPLC. The remaining sample was hydrolyzed. Since LA was the dominant PUFA in the liposome preparations no HETE-isomers were analyzed.

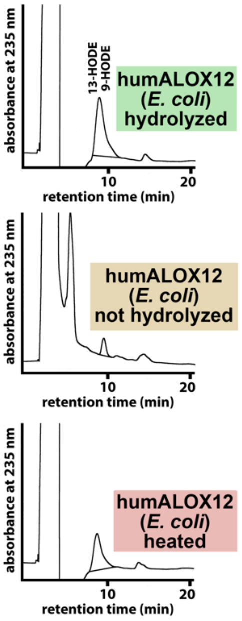

Figure S12. Liposome oxygenase activity of pure recombinant human ALOX15 expressed in Sf9 cells. Human ALOX15 was expressed as recombinant N-terminal his-tag fusion protein in Sf9 cells. The his-tag fusion proteins were purified by affinity chromatography on Ni-agarose. The ALOX15 containing elution fractions were pooled, desalted and aliquots of the pure enzyme preparations were employed to assay the liposome oxygenase activity. Heat inactivation was induced by heating the enzyme preparation for 5 min at 90° C. For the non-hydrolyzed samples an aliquot of the non-hydrolyzed lipid extract was directly injected to HPLC. The remaining sample was hydrolyzed.

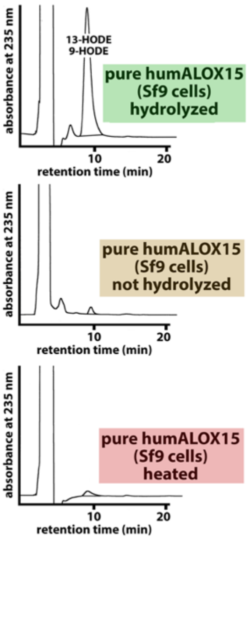

Figure S13. Alternative docking poses of 1-stearoyl-2-arachidonoyl-phosphatidylcholine (SAPC) at the active site of rabbit ALOX15. Molecular docking studies were carried out for the binding of SAPC at the active site of rabbit ALOX15 as described in detail in the STAR method section and three alternative docking poses were identified: i) *Pose 1:* The phosphocholine head group along with sn-2 unsaturated chain was positioned close to the iron atom, while the saturated sn-1 acyl chain extended out of the active site. ii) *Pose 2:* The unsaturated fatty acyl chain (sn-2) was oriented near the iron, with the saturated chain (sn-1) extending outside the binding pocket. iii) *Pose 3:* Both the saturated (sn-1) and unsaturated (sn-2) chains were located near the iron, with the C13 atom of the unsaturated chain positioned in close proximity to the iron centre, while the phosphocholine head group remained solvent-exposed. Almost identical docking poses were obtained when the SAPC was docked into the active site of human ALOX15B.

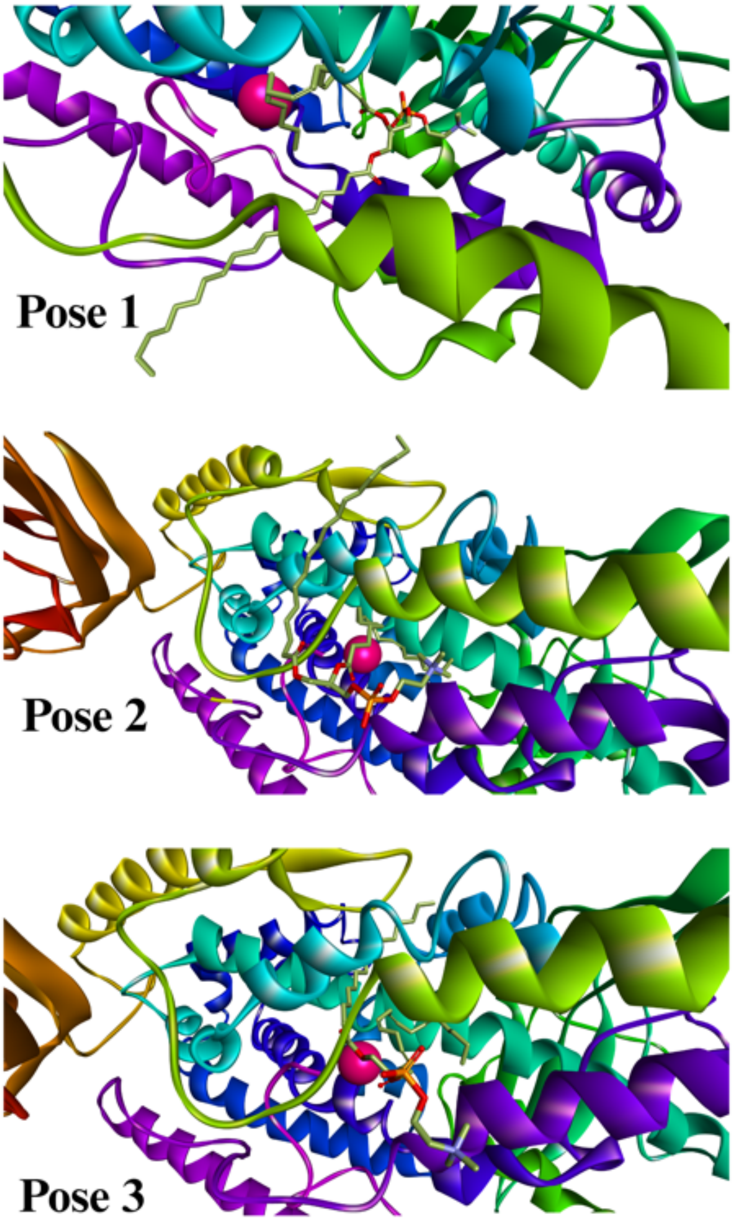

Figure S14. Mitochondrial membrane oxygenase activity of pure recombinant human ALOX15 expressed in Sf9 cells. Purified human recombinant ALOX15 was incubated in vitro with mitochondrial membranes for 15 min. After lipid extraction an aliquot of the extract was analyzed by RP-HPLC for the presence of conjugated dienes. The remaining lipid extract was hydrolyzed and an aliquot of the hydrolysate was also analyzed. Heat inactivation was induced by heating the enzyme preparation for 5 min at 90° C.

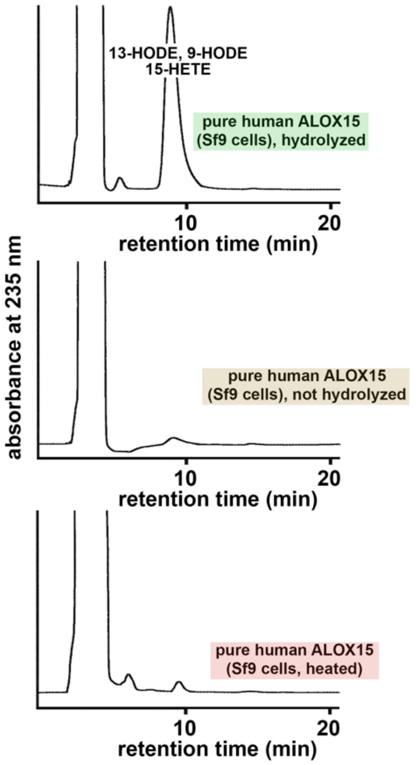

Figure S15. PUFA composition of erythrocyte ghosts. Erythrocyte membranes were prepared as described in the STAR Method section, the total membrane lipids were extracted and the ester lipids were hydrolyzed under alkaline conditions. Aliquots of the hydrolysates were injected into RP-HPLC for analysis of the major free PUFAs.

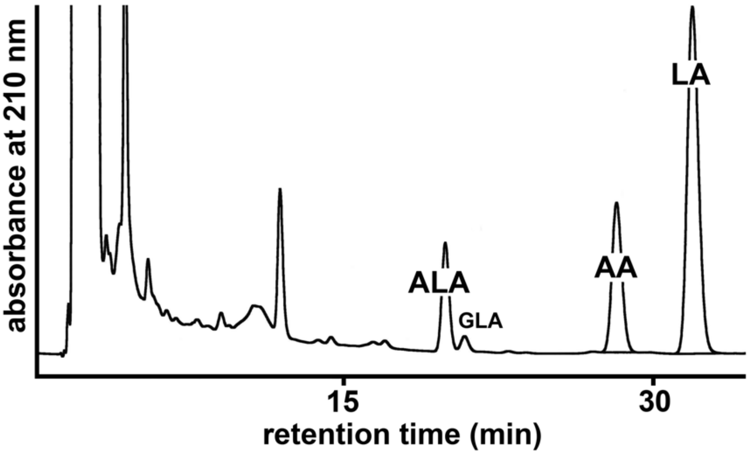

Figure S16. PUFA composition of human LDL and HDL. The two lipoprotein classes were prepared by sequential ultracentrifugation as described in the STAR Method section. The total lipids were extracted, the extracts were hydrolyzed under alkaline conditions. Aliquots of the hydrolysates were injected into RP-HPLC for analysis of the major free PUFAs. * unknown compound.

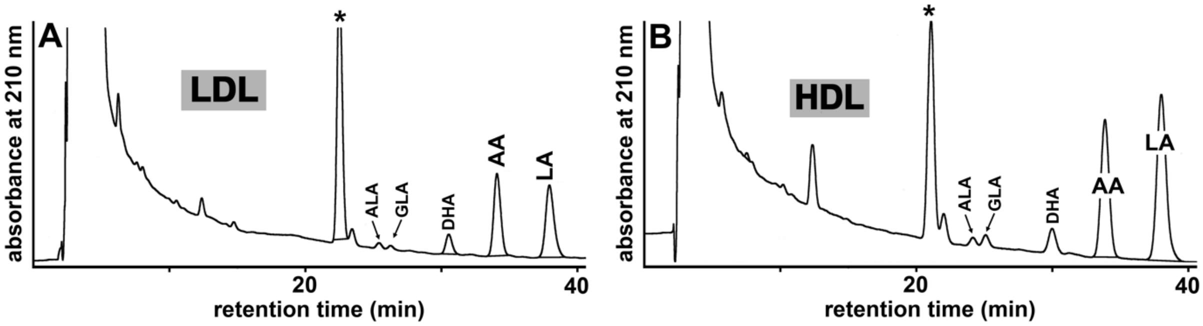

Figure S17. Original immunoblot used for the construction of Figure 1A.

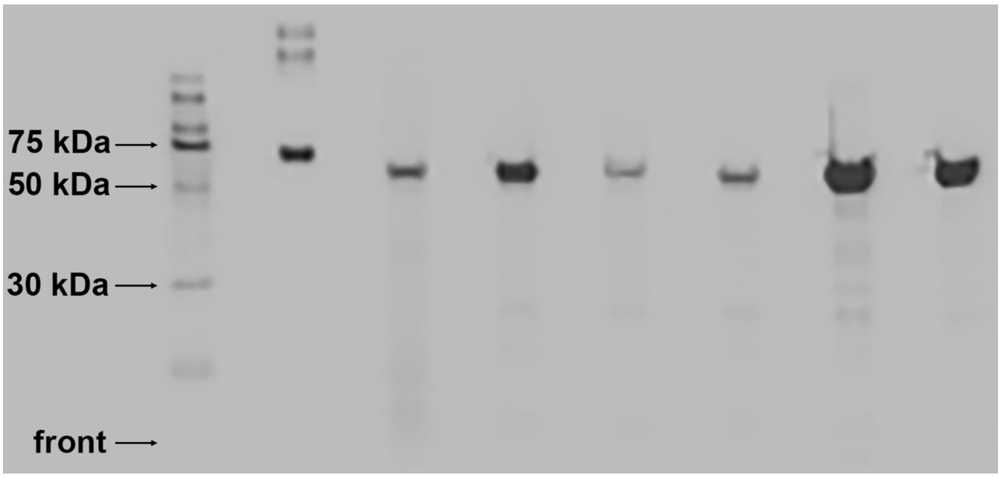

Table S1: Mammalian ALOX isoforms lack erythrocyte ghost oxygenase activity. Mammalian ALOX isoforms were expressed as N-terminal His-tag fusion proteins and aliquots of the cellular lysate supernatants representing identical AA oxygenase activities were incubated for 15 min with erythrocyte membranes (0.5 ml PBS, final membrane protein concentration 1 mg/ml). After hydroperoxide reduction the total membrane lipids were extracted. Extracts were hydrolyzed and the hydrolysates were analyzed by RP-HPLC following the absorbance at 235 nm (oxygenated PUFAs) and 210 nm (non-oxygenated PUFAs). OH-PUFA / PUFA ratios were calculated as suitable readout parameter for the degree of oxygenation of the membrane lipids. 3-4 independent incubations were run and the data were evaluated with the Student's t-test.

Enzyme	OH-PUFA / PUFA ratio (%)	Significance (vs. no enzyme)
No enzyme (PBS)	0.86 ± 0.14	-
Human ALOX15	-0.01 ± 0.01	***
Mouse Alox15	0.12 ± 0.09	**
Human ALOX15B	0.04 ± 0.33	**
Mouse Alox15b	0.17 ± 0.12	***
Human ALOX12	0.19 ± 0.10	***

These data indicate that the OH-PUFA / PUFA ratios in all enzyme incubations were lower than the value of the no-enzyme control incubation. Thus, the recombinant enzymes did actually protect the membrane ester lipids from ALOX-independent oxidation reactions.

Table S2. Mammalian ALOX isoforms do not induce hemolysis when incubated with mammalian ALOX preparations. Mammalian ALOX isoforms were expressed as N-terminal His-tag fusion proteins and aliquots of the cellular lysate supernatants representing identical AA oxygenase activities were incubated for 2 h with erythrocyte membranes (0.5 ml PBS, final membrane protein concentration 1 mg/ml). After the incubation period the degree of hemolysis was quantified by measuring the hemoglobin concentration (light absorbance at 410 nm) in the cell-free supernatant. 3-4 independent incubations were run for each enzyme and the data were evaluated with the Student's t-test. For total (100 %) hemolysis the erythrocytes were incubated for 45 min in 1 ml of ice-cold water. After the incubation period, membrane fragments were removed by centrifugation and the hemoglobin content in the stroma-free osmotic hemolysis supernatant was quantified by measuring the absorbance at 410 nm. PBS control: Instead of ALOX preparation a corresponding volume of PBS was added to the red blood cell suspension. E. coli control: Instead of the ALOX preparation a corresponding volume of E. coli lysis supernatant was added which was prepared from E. colic cells that were transformed with an empty expression plasmid. Sf9 cell control: Instead of the human ALOX15 preparation a corresponding volume of Sf9 cell lysate supernatant was added to the hemolysis sample, which were obtained from Sf9 cells that had been transfected with a non-recombinant baculovirus.

Sample	Time (h)	Absorbance at 410 nm	%	Time (h)	Absorbance at 410 nm	%
Osmotic hemolysis		21.220 ± 2.174	100.00		21.220 ± 2.174	100.00
PBS control	0.25	0.204 ± 0,023	0.96	4	0,204 ± 0,023	0.96
E. coli control	0.25	0,122 ± 0.015	0.57	4	0,122 ± 0,015	0.58
Sf9 cell control	0.25	0.129 ± 0.011	0.61	4	0,129 ± 0,011	0.61
Human ALOX15	0.25	0.116 ± 0,012	0.55	4	0,116 ± 0,012	0.55
Mouse Alox15	0.25	0,123 ± 0.091	0.58	4	0,123 ±0,091	0.58
Human ALOX15B	0.25	0.101 ± 0.018	0.48	4	0.101 ± 0.018	0.48
Mouse Alox15b	0.25	0,194 ± 0,029	0.92	4	0.194 ± 0.029	0.92
Human ALOX12	0.25	0,193 + 0.008	0.91	4	0.193 ± 0.008	0.91

The data shown in Table S2 clearly indicate that neither of the ALOX isoforms induced significant hemolysis under our experimental conditions. This finding is consistent with the absence of ALOX products in the erythrocyte membrane lipids after ALOX incubation (see **Table S1**).

Methodological supplement

1. Chemicals

The chemicals used in this study were purchased from the following vendors and the purposes of their use are specified in **Table S3**.

REAGENTS	SOURCE	IDENTIFIER	PURPOSE
**CHEMICALS**			
**Buffers, solutions and additives**			
Dubelco phosphate-buffered saline (DPBS)	PAN Biotech GmbH, Aidenbach, Germany	P0436500	Activity assays
Sodium borohydride	Sigma-Aldrich, St. Louis, USA	16940-66-2	Activity assays
Nitrocellulose membrane	Serva GmbH, Heidelberg, Germany	71223-01	Immunoblotting
Water (HPLC grade)	Fisher Scientific, New Hampshire, USA	W0106/17	Hemolysis experiment
Isopropyl-β-thiogalactopyranoside (IPTG)	Carl Roth GmbH, Karlsruhe, Germany	206-703-0	Enzyme preparation
Ethylenediaminetetraacetic acid (EDTA)	AppliChem, GmbH, Darmstadt, Germany	A3145,1000	Enzyme preparation
Potassium bromide (KBr)	Merck KG, Darmstadt, Germany	221864	Lipoprotein preparation
Soy phospholipid mixture	Avanti Polar Lipids, Birmingham, USA	690050P	Liposome preparation
Bovine heart cardiolipin	Sigma-Aldrich, Saint Louis, USA	SRE0029	Liposome preparation
Tris(hydroxymethyl)amino-methane (Tris)	Sigma-Aldrich, Saint Louis, USA	9210-OP	Lipoprotein preparation
Dithiothreitole (DDT)	Sigma-Aldrich, Saint Louis, USA	212-332-5	Endoplasmic membrane preparation
Agarose	SERVA GmbH, Heidelberg, Germany	11404.03	DNA electrophoresis
Insect XPRESS Medium	Biozym GmbH, Hessisch Oldendorf, Germany	881172	Enzyme expression
**Cells and animals**			
Sf9 cells	ThermoFisher, Schwerte, Germany	11496015	Enzyme preparation

E. coli, strain Rosetta2 DE3 pLysS	Novagen Darmstadt, Germany	71403-3	Enzyme preparation
Alox15$^{-/-}$ mice	Charles Rivers. Labs, Wilmington, USA	**(56)**	In vivo Alox15 activity assay
Alox15 knock-in mice	own production	**(57)**	In vivo ALOX15 activity assay
Transgenic aP2-ALOX15 mice	own production	**(70)**	In vivo ALOX15 activity assay
**Organic solvents**			
Acetonitrile (HPLC pure)	Fisher Scientific, New Hampshire, USA	10660131	RP-HPLC
Acetic acid	Fisher Scientific, New Hampshire, USA	10666821	RP-HPLC
Methanol	Fisher Scientific, New Hampshire, USA	15654570	NP/CP-HPLC
n-Hexane	Fisher Scientific, New Hampshire, USA	10703611	NP/CP-HPLC
2-Propanol	Fisher Scientific, New Hampshire, USA	15673990	NP/CP-HPLC
Ethanol	Fisher Scientific, New Hampshire, USA	10542382	NP/CP-HPLC
Chloroform	Fisher Scientific, New Hampshire, USA	10293850	NP/CP-HPLC
**Fatty acid substrates (PUFAs)**			
Arachidonic acid (AA)	Cayman Chem, Ann Arbor, USA	90010	Activity assays
Linoleic acid (LA)	Cayman Chem, Ann Arbor, USA	90150	Activity assays
alpha-Linoleic acid (ALA)	Cayman Chem, Ann Arbor, USA	90210	Activity assays
gamma-Linoleic acid (GLA)	Cayman Chem, Ann Arbor, USA	90220	Activity assays
**Eicosanoids**			
15*S*-HETE	Cayman Chem, Ann Arbor, USA	34720	Product analysis (HPLC)
15*R*-HETE	Cayman Chem, Ann Arbor, USA	34710	Product analysis (HPLC)

12*S*-HETE	Cayman Chem, Ann Arbor, USA	34570	Product analysis (HPLC)
12*R*-HETE	Cayman Chem, Ann Arbor, USA	34560	Product analysis (HPLC)
8*S*-HETE	Cayman Chem, Ann Arbor, USA	34360	Product analysis (HPLC)
8*R*-HETE	Cayman Chem, Ann Arbor, USA	34350	Product analysis (HPLC)
5*S*-HETE	Cayman Chem, Ann Arbor, USA	34230	Product analysis (HPLC)
5*R*-HETE	Cayman Chem, Ann Arbor, USA	34225	Product analysis (HPLC)
13*S*-HODE	Cayman Chem, Ann Arbor, USA	38610	Product analysis (HPLC)
13*R/S*-HODE	Cayman Chem, Ann Arbor, USA	38600	Product analysis (HPLC)
9*S*-HODE	Cayman Chem, Ann Arbor, USA	38410	Product analysis (HPLC)
9*S/R*-HODE	Cayman Chem, Ann Arbor, USA	38400	Product analysis (HPLC)
13S-HOTrE	Cayman Chem. Ann Arbor, USA	39620	Product analysis (HPLC)
**Human lipoproteins**			
Human low-density lipoprotein	Sigma-Aldrich, Saint Louis, USA	437644	LDL oxygenase assay
Human high-density lipoprotein	Sigma-Aldrich, Saint Louis, USA	437641	HDL oxygenase assay
**Enzymes, antibiotics and plasmids**			
SalI	ThemoFisher, Schwerte, Germany	GD0644	Enzyme expression
HindIII	ThemoFisher, Schwerte, Germany	IVGN0168	Enzyme expression
DNA ligase	ThemoFisher, Schwerte, Germany	EL0013	Enzyme expression
Ampicillin (sodium salt)	Carl Roth GmbH, Karlsruhe, Germany	K029.2	Enzyme expression

Kanamycin sulfate	Carl Roth GmbH, Karlsruhe, Germany	T832.3	Enzyme expression
Chloramphenicol	Carl Roth GmbH, Karlsruhe, Germany	3886.2	Enzyme expression
Protino Ni-NTA agarose	Macherey-Nagel GmbH, Düren, Germany	745200.120	Enzyme purification
Expression plasmid pET28b	Novagen-Merck, Darmstadt, Germany	69865	Enzyme expression
**Kits and DNA primers**			
Enpresso® B kit	Enpresso GmbH, Berlin, Germany	-	Enzyme expression
Bac-to-Bac baculovirus expression system	ThermoFisher, Schwerte, Germany	10359016	Enzyme preparation
Cloning and sequencing primers	BioTez Berlin GmbH, Berlin, Germany	-	Enzyme preparation
SERVALight Polaris CL HRP WB Kit	SERVA GmbH, Heidelberg, Germany	42584.02	Immunoblotting
Protein quantification kit	AppliChem, GmbH, Darmstadt, Germany	A6932,0500	Protein quantification
**Other reagents**			
Anti-6xHis-tag monoclonal antibody	ThemoFisher, Schwerte, Germany	MA1-21315	Immunoblotting
BlueBlock immunoblotting blocking solutions	SERVA GmbH, Heidelberg, Germany	42591.01	Immunoblotting
30% SDS solution	Biorad Labs Inc., Hercules, USA	161056	Protein electrophoresis
TEMED solution	Carl Roth GmbH, Karlsruhe, Germany	2367.1	Protein electrophoresis

2. Devices

The devices used in this study were purchased from the following vendors and the purposes of their use are specified in **Table S4**.

DEVICES	SOURCE	PURPOSE
Shimadzu binary gradient HPLC system	Shimadzu Deutschland GmbH, Duisburg, Germany	RP-HPLC

CTO-20 AC column oven	Shimadzu Deutschland GmbH, Duisburg, Germany	RP-HPLC
SIL-20AC auto-injector	Shimadzu Deutschland GmbH, Duisburg, Germany	RP-HPLC
LC-20AD pums	Shimadzu Deutschland GmbH, Duisburg, Germany	RP-HPLC
DGU-20A3 degaser	Shimadzu Deutschland GmbH, Duisburg, Germany	RP-HPLC
SPD-M20A diode array detector	Shimadzu Deutschland GmbH, Duisburg, Germany	RP-HPLC
Shimadzu isocratic HPLC system	Shimadzu Deutschland GmbH, Duisburg, Germany	NP/CP-HPLC
SPD-40 variable wavelength detector	Shimadzu Deutschland GmbH, Duisburg, Germany	NP/CP-HPLC
LC40 pump	Shimadzu Deutschland GmbH, Duisburg, Germany	NP/CP-HPLC
Shimadzu 1900i uv/vis spectrophotometer	Shimadzu Deutschland GmbH, Duisburg, Germany	HPLC calibration curves
TCC-100 temperature controler	Shimadzu Deutschland GmbH, Duisburg, Germany	HPLC calibration curves
Sonorex Super RK 512H ultrasonic bath	Bandelin Electronic GmbH, Berlin, Germany	Fatty acid solubilization
Branson W-250P tip sonifyer	Heineman, Schwäbisch-Gmünd, Germany	Enzyme preparation
Mini-Extruder	Avanti Polar Lipids, Birmingham, USA	Liposome preparation
PC membranes	Avanti Polar Lipids, Birmingham, USA	Liposome preparation
Beckman LE80K Ultracentrifuge	Beckman, Palo Alto, USA	Lipoprotein preparation
Eppendorf Biophotometer plus	Eppendorf SE, Hamburg, Germany	Hemolysis assay
FUJIFILM Luminescent Image Analyzer LAS-1000plus	Fujifilm Europe GmbH, Düsseldorf, Germany	Western blot quantification
Infors Multitron incubator	Infors HT, Einsbach, Germany	Enzyme preparation
