# Supplementary material for: Reactivity of mammalian lipoxygenases (ALOX isoforms) with phospholipids, biomembranes and lipoproteins
Source: Commun Biol. 2026 Jun 8;9:774. doi: 10.1038/s42003-026-10233-9 (PMC13246792; doi:10.1038/s42003-026-10233-9)
Supplement: Supplementary file 2 — Description of Additional Supplementary Files [file 42003_2026_10233_MOESM2_ESM.pdf]

Description of Additional Supplementary Files

File name: Supplementary Data 1

Description: This file provides the experimental raw data used for construction of the bar diagrams shown in Figs.1-7.

File name: Supplementary Data 2

Description: This file provides the experimental raw data used for construction of the bar diagram shown in Fig.8.

File name: Supplementary Movie 1

Description: MD simulation of the ALOX15-SAPC complex.

File name: Supplementary Movie 2

Description: MD simulation of the ALOX15B-SAPC complex.
