# Supplementary material for: Reactivity of mammalian lipoxygenases (ALOX isoforms) with phospholipids, biomembranes and lipoproteins
Source: Commun Biol. 2026 Jun 8;9:774. doi: 10.1038/s42003-026-10233-9 (PMC13246792; doi:10.1038/s42003-026-10233-9)
Supplement: Supplementary file 8 — Transparent Peer Review file [file 42003_2026_10233_MOESM8_ESM.pdf]

nature portfolio

Peer Review File

Reactivity of mammalian lipoxygenases (ALOX isoforms) with phospholipids, biomembranes and lipoproteins.

Corresponding Author: Professor Hartmut Kühn

This file contains all reviewer reports in order by version, followed by all author rebuttals in order by version.

Version 0:

Reviewer comments:

Reviewer #1

(Remarks to the Author)
The manuscript investigated the reactivity of mammalian ALOX isoforms with ester lipids in *in vitro* and in *in vivo* systems, and studied the structure of the enzyme-substrate complexes by docking studies and molecular dynamics simulations. The activities of ALOX isoforms using free PUFAs as substrates have been well studied, however, there were no systematic studies on activities using PUFA containing phospholipid as substrates before. Overall, the experiments are well designed, and the findings provide novel insights into roles of ALOX isoforms. The topic is of interest to the readers of this journal, and also to a wider field.

Major comments
1. In the study of liposome oxygenase activities (Figure 2), the authors used liposomes mainly containing LA, and almost no AA. Since the ALOX isoforms have either preference for LA or AA, the fatty acid composition of liposomes may affect activities. Is it possible to perform additional studies using liposomes mainly containing AA?
2. In the substrate docking studies and molecular dynamic simulations, the authors studied rALOX15 and hALOX15B. In the other studies, mALOX15 and hALOX15 were studied, however rALOX 15 was not studied. Is it possible to add information on why the authors selected rALOX15?
3. In the study using genetically modified mice (Figure 8), the authors suggested a possibility that plasma lipids contain PUFA containing phospholipids, which have been oxygenated by intracellular ALOX15. The authors studied products of free AA, LA, EPA, and DHA, so the data might be insufficient to support the hypothesis. Is there a difference in the amount of oxidized phospholipids?
4. In the "Animals and other research organisms" section of the reporting summary, it was stated that no experiments were carried out on life animals or on humans. However, in the materials and methods section of the paper, it was written that experiments were performed with animals (mouse and beef) and human blood. Is it possible to provide details of ethical approval?

Minor comments
1. There are few typographical errors in the manuscript (e.g., page 4 line87, line91).
2. The figure is incorrectly referenced in page 9, line 235.
3. Abbreviations are used inconsistently between the figures and the main text.

Reviewer #3

(Remarks to the Author)
This research investigates how various human and mouse lipoxygenase (ALOX) isoforms interact with complex ester lipids found in biomembranes and lipoproteins. While most of these enzymes typically prefer free fatty acids, the study found that human ALOX15, ALOX15B, and mouse mAlox15 are uniquely capable of directly oxygenating complex lipids without the need for helper proteins or external enzymes. In contrast, human ALOX12 and mouse mAlox15b exhibited significantly limited activity in these environments. Through a combination of molecular docking simulations and in vivo mouse models, the researchers confirmed that ALOX15 isoforms maintain their reaction specificity even when attacking membrane structures. The article highlights some interesting findings regarding these family of proteins and raises boarder questions into their activity within cellular biology and is relevant for publication.

However, some minor corrections are required and some clarity over certain concepts and results can help enhance the quality of the manuscript.

• Although this article provided critical insights into these enzymes' activities, the discussion was minimal to highlight how that might contribute to the pathogenesis of inflammatory, neurological, and hyperproliferative diseases, a concept introduced in the abstract but not discussed later. In addition, how this paper contributes to future studies and in broad the scientific community can be highlighted. To some extent, the discussion read like results and lacked critical analysis with current literature as well.

• Line 38, 40 and 42: human ALOX15, mALOX15 and human ALOX15B were mentioned. One can consider nALOX15 to be the abbreviation for mammalian ALOX, refereeing to the inclusion of all mammals. Maybe, use the term mouse Alox15 instead of mALOX15 so that you are consistent in the abstract and later use the assigned abbreviations throughout the body of the paper.

• Line 44: in vitro should be italic

• Line 56: Aloxe12 gene was mentioned. Is this the correct gene and spelling?

• Line 83, Section: Recombinant expression and functional characterization of mammalian ALOX isoforms.
Proteins expressed in Sf9 cells in comparison to E.coli will undergo post-translational modification like glycosylation. Whittington C et.al 2025 have recently shown glycosylation to impact the structure and functional activity of fungal LOXs.

Is there a possibility that having only one of the proteins (hALOX15) expressed in a eukaryotic system instead of the prokaryotic system may have had any impact on its activity?

• Figure 1B: The humanALOX15B AA oxygenase activity is shown as 2, 20. Is that correct?

• Line 119: Add space between detected in and correct the spelling of incubatios.

• Figures S7-S12: The x-axis ranges from 0-20 whereas in Figure 2D and F it is above 30. It would be nice to have them the same range for better comparison and understanding. As in some of the supplementary Figures, there seems to be presence of peaks that felt like got omitted due to the shorter x-axis.

• Line 147: Mentioning the difference in size between the liposomes in this section, might enhance the read.

• Line 172: Numbering of the figure is incorrect. Should be 1T.

• Line 179, section: Substrate docking studies and molecular dynamic simulations
The substrate binding pocket of mammalian ALOX isoforms is traditionally described as a narrow 'boot-shaped' cavity optimized for free fatty acids. How does the pocket accommodate the bulky stearoyl (C18:0) chain and the phosphocholine head group simultaneously without significant steric clashing?
What are the binding affinities and binding energies calculated between the three different poses and how does that compare to Free AA.
Can you provide an explanation as to why Pose 3 was selected and not the others?

• Figure 4: In Figure 4B, amino acids that might be interacting with the polar head group was shown. However, no such interactions or amino acid positioning near the polar head group in hALOX15B_SACP (Fig 4D) is shown or mentioned. It might be worthwhile to mention this or show this in Fig 4D.

• Line 199: His361 is mentioned. Is this correct?

• Line 200: Ile412 is mentioned. Is this the correct amino acid?

• Line 222: This network stabilizes the position of the polar head group and thus, of the entire phospholipid molecule at the active site of the enzyme.

The simulations do complement the experimental results to support the theory of possible interaction between the phospholipids and the enzyme which might then explain its liposomal oxygenase activity.

However, it does not account for the membrane effect and more importantly how does the phospholipid enter from the bilayer so deep into the pocket of the protein? Some discussion around this will improve the read.

• Line 233: LA was the major PUFA present in these membranes since its molar extinction
coefficient at 210 nm was about 4-fold lower than that of AA.

Better to revision this sentence. In its current structure, it reads like LA is the major PUFA which implies that it is present in greater concentration however from Figure 5C, AA shows a larger peak in comparison to LA. Given that LA has a lower

extinction coefficient I understand the rationale behind the statement but might benefit with better clarification.

• Line 235: Fig.5C vs 5D is mentioned. I think it should be 5B vs 5D.

• Line 256: Fig 1R+S is mentioned. Is this correct.

• Figure 6F: please check for spelling errors.

• Line 344 and 345: Fig.8F, G, H and Fig.8I, J, K are mentioned. Is that correct. Shouldn't it be (8F, H, J) and (8G, I, K).

• Line 354: Figure 8M, N, O, P is mentioned. Is that correct. Also, Fig and Figure are used. Might be better to be consistent.

• Line 375, section: Reaction specificity of mAlox15b with complex substrates is variable

Can differences in the packing order and lipid structural composition between the different membranes impact the activity of the enzymes and can that in turn influence the type and ratio of oxygenated products?

• Line 408: Moreover, their AA oxygenase activities very compromised (66-68). Please revise sentence.

• Line 437: Preparation of small and large liposomes
Liposomes are known to aggregate and fuse over time to form larger vesicles even if stored in the presence of inert gas. Sometimes, it is advisable to extrude the mixture regularly before use to ensure size uniformity. Where the liposomes characterized in any way for example DLS analysis?

• Figure 2J, shows a schematic representation of the interaction of ALOX with liposomes. One easy way to see if interaction of ALOX disrupts the lipid structural integrity of the liposomes can be studied by the use of liposomes encapsulating fluorescent dyes. Any interaction of the ALOX with the lipids would result in changing the orientation of the lipids which might result in membrane disruptions to release the dye from the liposomes into the solution that can be detected using fluorescent spectroscopy.

• Line 464: From the homogenates mitochondria were prepared by sequential differential centrifugation and mitochondria…

Prepared might not be the correct terminology to use.

• Line 499: Human LDL and HDL have been previously reported to function as substrates
for rabbit ALOX15.
Needs Referencing.

• Line 516 and 518: Correct spelling of butter to buffer.

• Line 573: Use of FCS in preparation of hALOX15 in Sf9 cells.

Is there any possibility of FCS having bovine lipooxygenases, that can be introduced into your lysate and contributed to the overall activity of hALOX15?

• Line 610: To quantify the free polyenoic fatty acid oxygenase activities of the different lipoxygenase preparations, aliquots of the cellular lysis supernatants (0.5 - 5 µL) were incubated.

WB was used to quantify the intensity of the different ALOX isoforms as seen in Figure 1A. Hence, different volumes of supernatant aliquots were used presumably to keep the concentration of the different his-tag ALOX isoforms constant for the assay. Is that correct?

If so, it would be nice to mention what was the final concentration of your his-tag ALOX isoforms used in the assay.

Since in all the other assays, the amount of supernatant used were normalized based on the activity seen in this assay. As a result, if different concentrations of his-tag recombinant proteins were used to determine their free fatty acid oxygenase activity, then direct comparisons cannot be made between the different isoforms without first proving that their activity is not concentration dependent.

• Line 644: can you provide details about the centrifugation.

• Lines from 636-655, 664-683 and 694-713, all have the same information. Might be better to state the protocol in the first time only and for the later sections just refer to the first section stating only the differences if any.

• Figure 1: mALOX15 is mentioned. Better to be consistent and use mAlox15.

• Please mention the number of replicates (n). Some Figure legends had that information while it was missing from others.

Version 1:

Reviewer comments:

Reviewer #1

(Remarks to the Author)
The authors have adequately addressed my previous comments.
The manuscript has been significantly improved.
In my opinion, the manuscript is now suitable for publication.

Reviewer #3

(Remarks to the Author)
I would like to thank the authors for incorporating the suggested changes and for clarifying specific points in their response and the revised manuscript.

After reviewing the revision and the authors' responses, I have no further comments. However, I noted a few remaining spelling errors that should be corrected. Additionally, please ensure that in vitro and in vivo are consistently italicized throughout the manuscript. I am happy to leave the final decision to the Editor's discretion.

Open Access This Peer Review File is licensed under a Creative Commons Attribution 4.0 International License, which permits use, sharing, adaptation, distribution and reproduction in any medium or format, as long as you give appropriate credit to the original author(s) and the source, provide a link to the Creative Commons license, and indicate if changes were made.
In cases where reviewers are anonymous, credit should be given to 'Anonymous Referee' and the source.
The images or other third party material in this Peer Review File are included in the article's Creative Commons license, unless indicated otherwise in a credit line to the material. If material is not included in the article's Creative Commons license and your intended use is not permitted by statutory regulation or exceeds the permitted use, you will need to obtain permission directly from the copyright holder.
To view a copy of this license, visit https://creativecommons.org/licenses/by/4.0/

CharitéCentrum 2 für Grundlagenmedizin

Charité | Campus Mitte | 10098 Berlin

Institut für Biochemie
Campus Charité Mitte
Chariteplatz 1, 10117 Berlin, Germany

Wiss. Direktoren: Prof. Dr. B. Eickholt,
Prof. Dr. M. Ralser

Bearbeiter: Prof. Dr. H. Kühn
Unser Zeichen:
Tel.: +49-30-450 528 040
e-mail: hartmut.kuehn@charite.de
www.charite.de/biochemie

Datum: 1.04.2026

Betrifft: response letter

Dear editor,

on behalf of all co-authors, we should like to extent our gratitude to the two expert reviewers for careful and critically reading our ms and for the helpful and constructive comments and suggestions. We have addressed all comments of the reviewers on the point-to point basis and we feel that revision did improve the quality of the manuscript. During revision we carried out additional experiments according to the advice of reviewer 1 and altered the ms accordingly. Moreover, we rephrased several parts of the ms, corrected misprints and spelling errors and modified some images (Fig. 1 and 4). We are submitting two different manuscript files. In the “marked-article.docx” file, which is submitted as supplemental file, the alterations introduced during revision are clearly labeled by yellow background. In the “article-file.docx”, which is for publication purpose, the labeling was removed. Otherwise, the two files are identical.

Reviewer 1

Comment 1 of reviewer 1: *The manuscript investigated the reactivity of mammalian ALOX isoforms with ester lipids in in vitro and in in vivo systems, and studied the structure of the enzyme-substrate complexes by docking studies and molecular dynamics simulations. The activities of ALOX isoforms using free PUFAs as substrates have been well studied, however, there were no systematic studies on activities using PUFA containing phospholipid as substrates before. Overall, the experiments are well designed, and the findings provide novel insights into roles of ALOX isoforms. The topic is of interest to the readers of this journal, and also to a wider field.*

Response of authors: We thank the reviewer for overall positive evaluation of our ms.

Comment 2 of reviewer 1: *In the study of liposome oxygenase activities (Figure 2), the authors used liposomes mainly containing LA, and almost no AA. Since the ALOX isoforms have either preference for LA or AA, the fatty acid composition of liposomes may affect activities. Is it possible to perform additional studies using liposomes mainly containing AA?*

Response of authors: We would like to thank the reviewer for bringing up the important point of chemical composition of the liposomes for the liposome oxygenase activity of mammalian ALOX-isoforms. However, the major aim of the liposome part of our study was to principally show that liposomes are substrates for different ALOX-isoforms since to the best of our knowledge, this has not been reported before. For these experiments we focused on linoleic acid containing liposomes since this fatty acid is the most abundant PUFA in most mammalian cells. Nevertheless, the reviewer is correct when saying that the liposome oxygenase activity of ALOX-isoforms may depend on the fatty acid composition of the liposomes. To address this point, we performed additional experiments and compared the oxygenation efficiency of different liposome preparation using the native rabbit ALOX15 as enzyme. Here we observed that not only the fatty acid composition (LA vs. AA vs. DHA) but also the phospholipid composition (PC vs. PE vs. PS) of the liposomes impacted the liposome oxygenase activity of this enzyme. These results were not really surprising but the data did open a number of interesting questions. However, to answer these questions more detailed additional experimentation is needed and this would clearly exceed the frame of the present study. To address this critical point of the reviewer we briefly discuss this issue in the Limitation section (page 15, line 419-417). Moreover, we initiated a follow-up project, in which we will systematically modify the lipid composition of the liposomes and test the oxygenation efficiency of different ALOX-isoforms.

Comment 3 of reviewer 1: *In the substrate docking studies and molecular dynamic simulations, the authors studied rALOX15 and hALOX15B. In the other studies, mALOX15 and hALOX15 were studied, however rALOX 15 was not studied. Is it possible to add information on why the authors selected rALOX15?*

Response of authors: This is an important point and we apologize for the confusion. In the revised version of the ms we clarified this issue (page 6/7, line 157-165). For the *in silico* docking studies and for MD simulations X-ray coordinates of the corresponding enzymes must be available. This is the case for rabbit ALOX15 and human ALOX15B but not for human ALOX15 and the mouse ALOX-isoforms used in our functional studies. However, rabbit ALOX15 shares a high degree of amino acid identity (some 86 %) with both human and mouse ALOX15 and thus, the 3D structure of rabbit ALOX15 may serve as suitable model for human and mouse ALOX15 orthologs.

Comment 4 of reviewer 1: *In the study using genetically modified mice (Figure 8), the authors suggested a possibility that plasma lipids contain PUFA containing phospholipids, which have been oxygenated by intracellular ALOX15. The authors studied products of free AA, LA, EPA, and DHA, so*

the data might be insufficient to support the hypothesis. Is there a difference in the amounts of oxidized phospholipids?

Response of authors: In Fig. 8 we show analytical data for the hydrolyzed plasma lipid extracts of different genetically modified mice. However, we also analyzed the non-hydrolyzed lipid extracts but here we only detected small amounts of oxygenated PUFAs. Taken together, this data indicates that the vast majority of the oxygenated PUFAs are present in the plasma ester lipids. We describe these findings in the revised version of the ms (page 11/12, line 305-314). We do however, admit that the presence of oxygenated PUFAs in the blood plasma ester lipids does not prove that intracellular ALOX15 did oxygenate the membrane ester lipids, which are subsequently transferred to the blood plasma. It might well be that the intracellular enzyme reacted with free PUFAs and that the free oxygenated PUFAs are subsequently incorporated into the membrane ester lipids which are then transferred to the blood plasma lipids. Both mechanistic scenarios are plausible but it is rather difficult to differentiate between the two pathways.

Comment 5 of reviewer 1: *In the "Animals and other research organisms" section of the reporting summary, it was stated that no experiments were carried out on life animals or on humans. However, in the materials and methods section of the paper, it was written that experiments were performed with animals (mouse and beef) and human blood. Is it possible to provide details of ethical approval?*

Response of authors: In this study we did not perform experiments on living animals nor on humans. However, we used beef hearts (obtained from the local slaughterhouse) to prepare submitochondrial particles, which were used as ALOX substrates (Fig. 5). Similarly, we used the liver of euthanized rats to prepare endoplasmic membranes (Fig. 6), prepared human LDL and HDL from blood plasma obtained from the local blood bank (Fig. 7) and employed the blood of sacrificed mice for our oxylipidome analyses (Fig. 8). However, according the German law no ethical approval is needed for these kinds of experiments.

Minor comments of reviewer 1: *a) There are few typographical errors in the manuscript (e.g., page 4 line87, line91). b) The figure is incorrectly referenced in page 9, line 235. c) Abbreviations are used inconsistently between the figures and the main text.*

Response of authors: We corrected the criticized misprints and figure referencing.

Reviewer 2

Comment 1 of reviewer 2: *This research investigates how various human and mouse lipoxygenase (ALOX) isoforms interact with complex ester lipids found in biomembranes and lipoproteins. While most of these enzymes typically prefer free fatty acids, the study found that human ALOX15, ALOX15B, and mouse mAlox15 are uniquely capable of directly oxygenating complex lipids*

without the need for helper proteins or external enzymes. In contrast, human ALOX12 and mouse mAlox15b exhibited significantly limited activity in these environments. Through a combination of molecular docking simulations and in vivo mouse models, the researchers confirmed that ALOX15 isoforms maintain their reaction specificity even when attacking membrane structures. The article highlights some interesting findings regarding these family of proteins and raises boarder questions into their activity within cellular biology and is relevant for publication.

Response of authors: We thank the reviewer for overall positive evaluation of our ms.

Comment 2 of reviewer 2*: Although this article provided critical insights into these enzymes' activities, the discussion was minimal to highlight how that might contribute to the pathogenesis of inflammatory, neurological, and hyperproliferative diseases, a concept introduced in the abstract but not discussed later. In addition, how this paper contributes to future studies and in broad the scientific community can be highlighted. To some extent, the discussion read like results and lacked critical analysis with current literature as well.*

Response of authors: Early on in the Discussion (page 14) of our ms we explicitly stated that ALOX catalyzed oxygenation of membrane ester lipids has previously been implicated in cell differentiation (ref. 21), erythropoiesis (ref. 25,26) and skin development (ref. 63, 64) and we give appropriate references for a number of these studies. We also report that oxygenation of membrane and lipoprotein lipids has previously been implicated in the pathogenesis of cardio-vascular and neurological diseases. This study was aimed at exploring the oxygenation of complex ester lipids by different ALOX isoforms but not at critically evaluating the different hypotheses on the biological relevance of these reactions. In response to the critical remark of the reviewer we modified the first paragraph of the discussion but refrained from critically discussing the pros and conts of the previously published hypotheses. We do agree with the reviewer that a summarizing critical discussion on ALOX catalyzed oxygenation of complex substrates would be useful for the future development of the field but such a critical review exceeds the frame of the present paper.

Comment 3 of reviewer 2*: Line 38, 40 and 42: human ALOX15, mALOX15 and human ALOX15B were mentioned. One can consider nALOX15 to be the abbreviation for mammalian ALOX, refereeing to the inclusion of all mammals. Maybe, use the term mouse Alox15 instead of mALOX15 so that you are consistent in the abstract and later use the assigned abbreviations throughout the body of the paper.*

Response of authors: We apologize for this mistake and followed the advice of the reviewer. We spelt out mAlox15 as mouse Alox15**.**

Comment 4 of reviewer 2*: Line 44: in vitro should be italic.*

Response of authors: Corrected as suggested by the reviewer.

Comment 5 of reviewer 2*: Line 56: Aloxe12 gene was mentioned. Is this the correct gene and spelling?*

Response of authors: When it was discovered this mouse lipoxygenase isoform was named e-12-LO, e-LOX1 and the corresponding gene *Alox12P1* or *Aloxe (*https://doi.org/10.1016/S0090-6980(02)00036-9). To synchronize the name of this lipoxygenase gene with those of the other mouse Alox genes (*Alox5, Alox15, Alox15b, Alox12, Alox12b, Aloxe3*) the name of this gene was later changed to *Aloxe12*.

Comment 6 of reviewer 2*: Line 83, Section: Recombinant expression and functional characterization of mammalian ALOX isoforms. Proteins expressed in Sf9 cells in comparison to E. coli will undergo post-translational modification like glycosylation. Whittington C et.al 2025 have recently shown glycosylation to impact the structure and functional activity of fungal LOXs. Is there a possibility that having only one of the proteins (hALOX15) expressed in a eukaryotic system instead of the prokaryotic system may have had any impact on its activity?*

Response of authors: Possible glycosylation of mammalian lipoxygenases is an interesting point, which has been discussed controversially in the past and this discussion was refreshed by the recent finding that the catalytic activity of a fungal lipoxygenase depends on glycosylation (doi: 10.1021/acs.biochem.4c00109). However, for the time being there is currently no conclusive evidence suggesting that mammalian ALOX isoforms are glycosylated nor that they require carbohydrates for their catalytic activities. In the early days of ALOX research it was reported that for rabbit ALOX15 about 5 % of its molecular weight was related to carbohydrates (doi: 10.1111/j.1432-1033.1979.tb13068) but later analyses indicated that this result was a preparation artifact. Human ALOX15 expressed in Sf9 insect cells is neither glycosylated (doi: 10.1016/0005-2760(93)90085-n).

Comment 7 of reviewer 2*: Figure 1B: The humanALOX15B AA oxygenase activity is shown as 2, 20. Is that correct?*

Response of authors: This misprint was corrected in the revised version of the ms.

Comment 8 of reviewer 2*: Line 119: Add space between detected in and correct the spelling of incubatios.*

Response of authors: This misprint was corrected in the revised version of the ms.

Comment 9 of reviewer 2*: Figures S7-S12: The x-axis ranges from 0-20 whereas in Figure 2D and F it is above 30. It would be nice to have them the same range for better comparison and understanding. As in some of the supplementary Figures, there seems to be presence of peaks that felt like got omitted due to the shorter x-axis.*

Response of authors: In Figure 2D + F we showed the long range of the x-axis since non-oxygenated PUFAs elute with retention times > 25 min. However, recording the chromatogram at 235 nm (detection of oxidized PUFAs) the x-axis region between 20 and 38 min is not productive and thus, we did not show this region in the supplemental control experiments (Figure S7-S12). The reviewer is probably referring to Figure S9 (lower trace, heated enzyme) when saying “that there seems to be the presence of peaks that felt like got omitted due to the shorter x-axis”. We went back to the original chromatograms of this sample and found that there is indeed a small peak eluting at around 21 min. However, the uv-spectrum of this peak did not indicate a conjugated diene. Thus, whatever this peak may represent, it is not a primary ALOX product. The major take-home messages of the control chromatograms are not impacted in any way by the presence or absence of additional unknown peaks in this region of the chromatogram. Thus, we would like to keep the supplemental images as they are.

Comment 10 of reviewer 2*: Line 147: Mentioning the difference in size between the liposomes in this section, might enhance the read.*

Response of authors: We follow the advice of the reviewer and give the size of the small and large liposomes in the text.

Comment 11 of reviewer 2*: Line 172: Numbering of the figure is incorrect. Should be 1T.*

Response of authors: This error was corrected.

Comment 12 of reviewer 2*: Line 179, section: Substrate docking studies and molecular dynamic simulations. The substrate binding pocket of mammalian ALOX isoforms is traditionally described as a narrow 'boot-shaped' cavity optimized for free fatty acids. How does the pocket accommodate the bulky stearoyl (C18:0) chain and the phosphocholine head group simultaneously without significant steric clashing? What are the binding affinities and binding energies calculated between the three different poses and how does that compare to Free AA. Can you provide an explanation as to why Pose 3 was selected and not the others?*

Response of authors: When we started this project the authors were also under the impression that the structures of the substrate binding pockets of mammalian ALOX isoforms were optimized to bind polyenoic fatty acids and this is still the case for mammalian ALOX5 and ALOX12 orthologs. However, human and rabbit ALOX15 as well as human ALOX15B are capable of oxygenating more complex ester lipids and thus, there must be enough space in the substrate binding pocket of these enzymes so that more bulky substrates such as phospholipids (this paper), endocannabinoids (doi: 10.1016/j.bbalip.2021.158918) as well PUFA-carnitine and PUFA co-enzyme A esters (doi: 10.1016/j.jlr.2025.100861) can bind. The binding energies for the three relevant docking poses were almost identical and pose 3 was selected because in this docking pose C13 of the arachidonic acid backbone was in close proximity to the enzyme-bound non-heme iron. This is an

essential precondition for the catalytic productivity of the enzyme substrate complex. In the revised version of the ms this is now explained in more detail (page 6-7, line 157-165).

Comment 13 of reviewer 2*: Figure 4: In Figure 4B, amino acids that might be interacting with the polar head group was shown. However, no such interactions or amino acid positioning near the polar head group in hALOX15B_SACP (Fig 4D) is shown or mentioned. It might be worthwhile to mention this or show this in Fig 4D.*

Response of authors: The authors are grateful to the reviewer for this observation. In the selected docking pose of the ALOX15B-SAPC complex, the polar head group of the substrate was not positioned in close proximity to any active site amino acid and thus, we did not show such interaction in Fig. 4D. However, during the MD simulation, the polar head group moved around and was finally stabilized by interacting with the side-chains of Arg429 and Gln425. This finding was described in the MD simulation section of the Results chapter. In response to the critical comment of the reviewer we modified Figure 4D (annotated the two critical amino acids Arg429 and Gln425) and also rephrased the legend to this figure.

Comment 14 of reviewer 2*: Line 199: His361 is mentioned. Is this correct?*

Response of authors: His361 is wrong and was corrected to His373 in the revised version of the ms. This point of criticism clearly indicates the ALOX expertise of the reviewer. Based on the X-ray coordinates of human ALOX15B (4NRE) His373, His378, His553 and the N-terminal Ile676 function as direct iron ligands. Amino acid numbering of this enzyme in the PDB dataset starts with Met1 although in the native protein Ala2 is the C-terminal amino acid. This may lead to confusion for non-expert readers but there is nothing one can do about it.

Comment 15 of reviewer 2*: Line 200: Ile412 is mentioned. Is this the correct amino acid?*

Response of authors: According to the 4NRE dataset Ile412 is correct.

Comment 16 of reviewer 2*: Line 222: This network stabilizes the position of the polar head group and thus, of the entire phospholipid molecule at the active site of the enzyme. The simulations do complement the experimental results to support the theory of possible interaction between the phospholipids and the enzyme which might then explain its liposomal oxygenase activity. However, it does not account for the membrane effect and more importantly how does the phospholipid enter from the bilayer so deep into the pocket of the protein? Some discussion around this will improve the read.*

Response of authors: This is an important question which cannot completely be answered at this stage of research. We think that the enzymes bind at the surface of the particles (liposomes, membranes, lipoproteins) and then “extract” a complete phospholipid molecule from the outer phospholipid layer. Such “extraction” may be possible when the substrate binding pocket offers a more

suitable hydrophobic environment than the phospholipid layer. Once the phospholipid molecule is bound in the substrate binding pocket it may be oxygenated and may subsequently be re-incorporated into the lipid layer. We visualize this mechanistic scenario for liposome oxygenation in Fig. 2J and explain it on page 5-6, lines 127-134) of the original ms. Similar mechanisms may be relevant for the oxygenation of biomembranes and lipoproteins.

Comment 17 of reviewer 2*: Line 233: LA was the major PUFA present in these membranes since its molar extinction coefficient at 210 nm was about 4-fold lower than that of AA. Better to revision this sentence. In its current structure, it reads like LA is the major PUFA which implies that it is present in greater concentration however from Figure 5C, AA shows a larger peak in comparison to LA. Given that LA has a lower extinction coefficient I understand the rationale behind the statement but might benefit with better clarification.*

Response of authors: When we discussed the chromatograms with non-lipid experts these colleagues often replied that according to our HPLC data AA is present in mitochondrial membranes in higher quantities than LA since the peak area of the AA peak was higher. However, according to our calibration curves AA has a 4-fold higher molar extinction coefficient than LA at 210 nm and thus LA is the dominant PUFA in these types of biomembranes. This fact requires an explanation since otherwise our data would conflict with previous reports on the PUFA composition of human LDL. We followed the advice of the reviewer and rephrased this paragraph to improve its legibility (page 8, lines 207-210).

Comment 18 of reviewer 2*: Line 235: Fig.5C vs 5D is mentioned. This should be 5B vs 5D.*

Response of authors: We corrected the wrong figure labeling.

Comment 19 of reviewer 2*: Line 256: Fig 1R+S is mentioned. Is this correct.*

Response of authors: Fig. 1R shows RP-HPLC of the AA oxygenation products formed by mouse Alox15b (8-HETE) and Fig. 1S shows the enantiomer composition of this product. (8S-HETE). Thus, our figure labeling is correct.

Comment 20 of reviewer 2*: Figure 6F: please check for spelling errors.*

Response of authors: The spelling error was corrected.

Comment 21 of reviewer 2: *Line 344 and 345: Fig.8F, G, H and Fig.8I, J, K are mentioned. Is that correct. Shouldn't it be (8F, H, J) and (8G, I, K).*

Response of authors: We corrected the faulty figure labeling in the text.

Comment 22 of reviewer 2: *Line 354: Figure 8M, N, O, P is mentioned. Is that correct. Also, Fig and Figure are used. Might be better to be consistent.*

Response of authors: We also corrected this figure labeling. For consistency we abbreviated all figures as Fig. in the text.

Comment 23 of reviewer 2: *Line 375, section: Reaction specificity of mAlox15b with complex substrates is variable. Can differences in the packing order and lipid structural composition between the different membranes impact the activity of the enzymes and can that in turn influence the type and ratio of oxygenated products?*

Response of authors: Although we cannot rule out that packing order and lipid composition of the different types of biomembranes may impact the variable reaction specificity of mouse Alox15b a more plausible explanation for this enzyme property is its unusual reaction specificity with free AA. This problem has been reported before (ref. 36) and here we added additional information to this phenomenon. However, the molecular basis for this enzyme property remains a matter of discussion.

Comment 24 of reviewer 2: *Line 408: Moreover, their AA oxygenase activities very compromised (66-68). Please revise sentence.*

Response of authors: We followed the advice of the reviewer and rephrased this sentence (page 14, line 394-395). It now reads: Moreover, the AA oxygenase activities of these two enzymes strongly depend on the reaction conditions **(66-68)** and thus, our normalization strategy would not work with them.

Comment 25 of reviewer 2: *Line 437: Preparation of small and large liposomes Liposomes are known to aggregate and fuse over time to form larger vesicles even if stored in the presence of inert gas. Sometimes, it is advisable to extrude the mixture regularly before use to ensure size uniformity. Where the liposomes characterized in any way for example DLS analysis?*

Response of authors: We did not routinely perform DLS analyses with our liposome preparations. However, for each experiment used freshly prepared liposome and these preparations were never stored longer than 8 hours. This information is now given in the Material and Method section (page 16, line 450-451).

Comment 26 of reviewer 2: *Figure 2J, shows a schematic representation of the interaction of ALOX with liposomes. One easy way to see if interaction of ALOX disrupts the lipid structural integrity of the liposomes can be studied by the use of liposomes encapsulating fluorescent dyes. Any interaction of the ALOX with the lipids would result in changing the orientation of the lipids which might result in membrane disruptions to release the dye from the liposomes into the solution that can be detected using fluorescent spectroscopy.*

Response of authors: This is an interesting and constructive suggestion that is worth to be explored in a follow-up project. In fact, we already initiated a number of experiments along this line to

investigate the structural and functional consequences of ALOX attack on liposomes. However, such experiments require a different methodological profile and these methods are currently established in the lab.

Comment 27 of reviewer 2: *Line 464: From the homogenates mitochondria were prepared by sequential differential centrifugation and mitochondria…Prepared might not be the correct terminology to use.*

Response of authors: We rephrased this sentence: It now reads: From the tissue homogenates the mitochondria were isolated by differential centrifugation (Fig. 16, line 458-459).

Comment 28 of reviewer 2: *Line 499: Human LDL and HDL have been previously reported to function as substrates for rabbit ALOX15. Needs Referencing.*

Response of authors: We included an appropriate reference (ref 31, page 18).

Comment 29 of reviewer 2: *Line 516 and 518: Correct spelling of butter to buffer.*

Response of authors: This is a funny typo that escaped our attention but also the attention of our electronic spell checker. The reviewer found it. Respect!!! We corrected this typo in the revised ms.

Comment 30 of reviewer 2: *Line 573: Use of FCS in preparation of hALOX15 in Sf9 cells. Is there any possibility of FCS having bovine lipoxygenases, that can be introduced into your lysate and contributed to the overall activity of hALOX15?*

Response of authors: We tested our FCS for the presence of ALOX15 by immunoblotting and activity assays but did not get any evidence for the presence of a functional ALOX isoform. Moreover, bovine ALOX15 constitutes an arachidonic acid 12-lipoxygenating ALOX15 ortholog. Thus, if the human ALOX15 preparation would be contaminated with bovine ALOX12 a different pattern of oxygenation products would be expected. In fact, our basic enzyme characterization (Fig. 1) excluded such contamination.

Comment 31 of reviewer 2: *Line 610: To quantify the free polyenoic fatty acid oxygenase activities of the different lipoxygenase preparations, aliquots of the cellular lysis supernatants (0.5 - 5 µL) were incubated. WB was used to quantify the intensity of the different ALOX isoforms as seen in Figure 1A. Hence, different volumes of supernatant aliquots were used presumably to keep the concentration of the different his-tag ALOX isoforms constant for the assay. Is that correct? If so, it would be nice to mention what was the final concentration of your his-tag ALOX isoforms used in the assay. Since in all the other assays, the amount of supernatant used were normalized based on the activity seen in this assay. As a result, if different concentrations of his-tag recombinant proteins were*

used to determine their free fatty acid oxygenase activity, then direct comparisons cannot be made between the different isoforms without first proving that their activity is not concentration dependent.

Response of authors: We did not normalize our enzyme preparations for the same amounts of recombinant ALOX protein but for the same arachidonic acid oxygenase activity instead. In other words, we prepared the lysate supernatants of E. coli and Sf9 cells and tested the arachidonic acid oxygenase activities of the different enzyme preparations. For this purpose, identical volumes of the lysate supernatants (2 µL) were employed. Next, we quantified the oxygenation products formed during a 3 min incubation period and this measure was used as readout parameter for the AA oxygenase activity of the different enzyme preparations. Finally, we calculated the lysate supernatant volumes, which correspond to the same arachidonic acid oxygenase activity. These volumes were then used in the liposome, the biomembrane and the lipoprotein oxygenase activity assays. In other words, if 5 µL of recombinant human ALOX15 produced 10 nmoles of oxygenation products during the 3 min incubation period but 5 µl of mouse Alox15 produced only 5 nmoles we added 10 µL of mouse Alox15 to the liposome activity assays. We feel that this activity-based normalization procedure is more suitable than ALOX-amount based normalization since the specific catalytic activities of the different enzymes are different. We explained our normalization procedure in more detail in the revised version of the ms (page 22, line 625-634).

Comment 32 of reviewer 2: *Line 644: can you provide details about the centrifugation.*

Response of authors: The centrifugation details are given in the revised version of the ms. (20,000 x g for 15 min at 4°C).

Comment 33 of reviewer 2: *Lines from 636-655, 664-683 and 694-713, all have the same information. Might be better to state the protocol in the first time only and for the later sections just refer to the first section stating only the differences if any.*

Response of authors: We follow the advice of the reviewer and describe the experimental details for the liposome oxygenase assay and refer to these details in the description of the membrane and lipoprotein oxygenase activity assays. Thus, the text of the revised ms is less redundant.

Comment 34 of reviewer 2: *Figure 1: mALOX15 is mentioned. Better to be consistent and use mAlox15.*

Response of authors: We corrected the figure according to the advice of the reviewer.

Comment 35 of reviewer 2: *Please mention the number of replicates (n). Some Figure legends had that information while it was missing from others.*

Response of authors: We went over all the figure legends and provided the requested information.

We hope that we have adequately addressed the critical remarks of the reviewers and that the revised version of the ms is now acceptable for publication.

Sincerely,

H. Kuhn, P. Aparoy

CharitéCentrum 2 für Grundlagenmedizin

Charité | Campus Mitte | 10098 Berlin

Institut für Biochemie
Campus Charité Mitte
Chariteplatz 1, 10117 Berlin, Germany

Wiss. Direktoren: Prof. Dr. B. Eickholt,
Prof. Dr. M. Ralser

Bearbeiter: Prof. Dr. H. Kühn
Unser Zeichen:
Tel.: +49-30-450 528 040
e-mail: hartmut.kuehn@charite.de
www.charite.de/biochemie

Datum: 20.04.2026

Betrifft: response letter

Dear editor,

on behalf of all co-authors, we should like to extent our gratitude to the two expert reviewers for careful and critically reading our ms and for their favorable recommendations. We have addressed minor comments of the reviewers on the point-to point basis in this response letter and modified the ms according to these comments and the editorial recommendations.

Reviewer 1

Comment of reviewer 1: *The authors have adequately addressed my previous comments. The manuscript has been significantly improved. In my opinion, the manuscript is now suitable for publication.*

Response of authors: We thank the reviewer for positive evaluation of the revised version of our ms.

Reviewer 2

Comment 1 of reviewer 2: I would like to thank the authors for incorporating the suggested changes and for clarifying specific points in their response and the revised manuscript. After reviewing the revision and the authors' responses, I have no further comments.

Response of authors: We thank the reviewer for positive evaluation of the revised version of our ms.

Comment 2 of reviewer 2: *However, I noted a few remaining spelling errors that should be corrected. Additionally, please ensure that in vitro and in vivo are consistently italicized throughout the manuscript. I am happy to leave the final decision to the Editor's discretion.*

Response of authors: We did our best to correct the spelling errors but for non-native speakers this is somewhat challenging. We also employed two different spell-checking programs but it might well be that some typos still remain. Here we ask kindly for editorial assistance. We also italicized the terms "*in silico*", "*in vitro*" and "*in vivo*" throughout the ms.

Editorial comments

We modified the text of the ms according to the editorial recommendations specified in the "Final revision instruction" table. The completed table will be submitted as a related manuscript file.

We hope that we have adequately addressed the critical remarks made by the two reviewers and the formal aspects brought up by the editor. We hope that the revised version of the ms is now acceptable for publication.

Sincerely,

H. Kuhn, P. Aparoy
